# Wet Carbonation of Industrial Recycled Concrete Fines: Experimental Study and Reaction Kinetic Modeling

**DOI:** 10.1021/acs.iecr.5c02835

**Published:** 2025-10-30

**Authors:** Z. Tabrizi, C. Rodriguez, E. Barbera, W. R. Leal da Silva, F. Bezzo

**Affiliations:** † CAPE-Lab−Computer-Aided Process Engineering Laboratory, Department of Industrial Engineering, University of Padova, 35131 Padova, Italy; ‡ FLSmidth Cement A/S, Green Innovation, 2500 Valby, Copenhagen Denmark; § Institute for Technical Chemistry, Karlsruhe Institute of Technology, 76344 Karlsruhe, Germany

## Abstract

Carbon dioxide mineralization via wet carbonation of industrial Recycled Concrete Fines (RCFs) offers a promising pathway for mitigating emissions in the cement industry, necessitating reliable kinetic models for technology scale-up. This work proposes a validated diffusion-based Shrinking Core Model describing the wet carbonation kinetics of RCFs. The model, based on parabolic diffusion law, is rigorously selected and calibrated among mineralization models in wet systems. Experimental results demonstrate a maximum carbonation efficiency of 0.81, corresponding to 95 kg CO_2_ uptake per tonne of RCFs, and acceptable compressive strength development when incorporating RCFs up to 10% in blended cement. Reaction rates showed a minimal temperature impact due to the offset between the CO_2_ solubility and diffusion through the product layer. Compared to Recycled Cement Paste (RCP) carbonation, higher diffusion coefficients are predicted, likely caused by looser product layer. Analysis highlights the importance of particle size and the CO_2_ partial pressure, providing insights for efficient scale-up.

## Graphical abstract

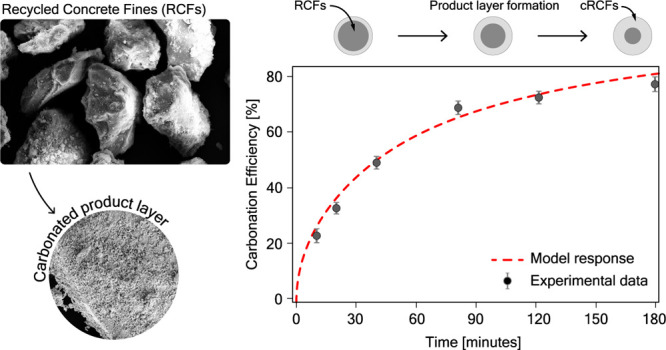

## 1 Introduction

The cement industry, often cited as contributing ∼5–8% of global anthropogenic CO_2_ emissions, has become a major focus of research into carbon capture, storage, and utilization (CCUS).^1–3^ Among these, CO_2_ mineralization has gained significant interest due to its dual role in decarbonization and valorization of industrial byproducts.^4^ By converting CO_2_ into calcium or magnesium carbonates (i.e., CaCO_3_ and MgCO_3_), this process offers permanent storage with minimal risk of re-release.^5,6^ Moreover, CO_2_ mineralization enhances the value of the supplementary cementitious materials (SCMs) by converting them into carbonated SCMs (cSCMs).^6^ Incorporating cSCMs into cement not only reduces the demand for clinker but also contributes to carbon sequestration. Among a wide range of SCMs, RCFs are of particular interest due to their enhanced pozzolanic reactivity upon carbonation, which improves the mechanical properties of blended cements and enables CO_2_ sequestration, contributing to more sustainable concrete materials.^7,8^

RCFs are considered emerging SCMs crucial for supporting the decarbonization of the cement industry.^9^ They are obtained during the recycling of construction and demolition of concrete and accounted for approximately 35% of the global generation of carbonatable solid materials in 2020, equivalent to 1.4 Gt/y.^10^ The recycling process involves collecting, crushing, sieving, and separating to produce aggregates, sands, and fines. Despite the challenge of complete separation of the fine fraction (i.e., cement paste) from aggregates and sands,^11^ the RCFs hold significant potential for CO_2_ sequestration, with ca. 70 to 300 kg CO_2_ per tonne of RCFs reported in the literature.^12,13^ The upper bound values are typically associated with laboratory-produced RCFs, which mainly represent pure hydrated cement paste; whereas the lower bound values relate to industrial RCFs due to their inherited natural carbonation during service life.^14^ If the entire volume of RCFs were carbonated and used as clinker replacement in cement, the carbonated RCFs (cRCFs) could offer a decarbonization potential of up to 0.39 Gt CO_2_/y, i.e., 15% of that from cement production.^10^ Various approaches have been proposed to produce cRCFs, including slurry grinding to refresh reactive surfaces,^15^ semiwet processes with additive salts to enhance CO_2_ dissolution,^16^ and hyper-gravity carbonation, which accelerates mass transfer and promotes rapid calcite and silica gel formation;^17^ nevertheless, wet carbonation remains the baseline for its simplicity, fast kinetics, and high efficiency, with emerging methods offering further improvements.^18^

Producing cRCFs and their subsequent use as SCM require optimally designed reactors, supported by kinetic modeling, lab-to-industrial scale-up, and precisely calculated residence time of species.^19^ Mineral carbonation kinetic models generally fall into two groups: surface activity models such as Surface Coverage Model,^20–24^ and diffusion-reaction resistance models such as the Basic and Modified Shrinking Core Models (BSCMs, MSCMs).^25^ The latter considers more physical variability and incorporates mechanisms such as product layer and gas–liquid film diffusivity,^26^ clogging, and diffusivity changes with reaction progress,^26–28^ and are distinguished by assumed particle geometry. While these approaches have described the carbonation of SCMs, studies on the carbonation of materials derived from concrete demolition sites remains limited. Existing studies have focused mostly on CaO-rich materials such as RCP or laboratory-produced RCFs, which predominantly consist of hydrated cement paste. These materials serve as a proxy for RCFs but do not account for the long-term environmental exposure of concrete. Thus, there exists a research gap in proposing accurate kinetic model(s) for the carbonation of industrial RCFs.

Carbonation modeling of laboratory-produced RCFs together with the effects of water-to-solid ratio has been carried out by Mehdizadeh et al. in a dry-pressurized autoclave using the surface coverage model.^29^ This model describes reaction progress as a result of surface deactivation, simplifying the material properties by treating the reaction contact area as the only variable.^22^ In another study, Mao et al. investigated RCP carbonation and used the Jander’s model to predict carbonation efficiency;^30^ which consists in a simpler diffusion-based model of Shrinking Core Models dominated by product layer diffusion. These models neither explicitly account for material properties beyond specific surface area or particle size nor include key process driving forces such as CO_2_ partial pressure and reaction temperature within the model structure as part of a first-principles approach which limits their applicability.

A more generalized form of Shrinking Core Models can account for some of the missed variabilities that are critical for application in realistic reactors. It considers the reaction as a quasi-steady process dominated by either diffusion through the liquid film around the particle, reaction on the surface, or, more relevantly, diffusion through the product layer.^25^ The latter can be interpreted as involving a time-varying diffusion coefficient^28^ or a parabolic diffusion law^27^ rather than the Fick’s law. All these formulations vary based on the initial particle geometry; thermal dependency is typically described using the Arrhenius’ law. Various carbonation systems, dry or wet, can influence the model by accounting for the solubility of CO_2_ in the solvent, rather than directly considering the CO_2_ partial pressure in diffusion and reaction.^26^ Applying such models to describe the carbonation of RCFs could offer a more comprehensive understanding of the influence of the raw material properties and gas phase composition.

Additionally, the process of discriminating between various predictive models, selecting the most representative one, and calibrating it to an acceptable level of accuracy—commonly referred to as model identification—has not been adequately addressed in the field of mineral carbonation. This gap is particularly critical given the limited availability of data and the deviations from idealized model assumptions often observed in such systems, which contribute to uncertainties in modeling. A rigorous approach to model identification can help address and reduce these uncertainties by applying the appropriate statistical metrics and numerical techniques.

In that light, this study aims to address the identified modeling gaps by proposing a predictive kinetic model for the wet carbonation of industrial RCFs, supported by experiments conducted in a semibatch reactor. The reacting RCFs and its carbonated counterpart (cRCFs) are characterized to confirm their suitability as SCMs and support the formulation of physically representative models. A rigorous approach has been employed to discriminate between candidate models and to calibrate the selected model under low-information conditions. The consistency between model-based parameters and experimental measurements serves as a basis for confirming the validity of modeling assumptions and discussing trade-offs between the influencing mechanisms considering the observed data. Furthermore, these insights are used to propose optimization pathways for the reaction process and guide the design of more informative kinetic experiments.

## 2 Material and Methods

### 2.1 Materials

Waste concrete was obtained after demolition of a structure with 100+ years of service in Denmark. In the field, the debris were screened and classified in coarse aggregate (4–20 mm) and sand (0–4 mm). Subsequently, the sand fraction was sieved in the laboratory to obtain industrial RCFs with a narrow particle size between 45 and 75 μm. For the carbonation experiments, demineralised water and commercial-grade CO_2_ (>99.9%) and N_2_ (99.6%) gases were used in all experiments.

### 2.2 Experimental Setup

A wet carbonation setup (Figure 1) was commissioned and used to perform the RCFs carbonation experiments. This apparatus was designed to allow for adjustments on gas composition and temperature control of both gas and liquid phases in the reactions. Experiments were carried out at the operational conditions of 25, 45, 65, and 85 °C, ambient pressure, and a solid-to-liquid mass ratio of 10% (corresponding to 20 g of RCFs) and samples were taken at 0, 10, 20, 40, 80, 120, and 180 min during the reaction time. A simulated gas mixture, comprising 15 vol % CO_2_ and 85 vol % N_2_ to represent emissions from clinker production in cement plants or from limestone calcination in lime kilns, was injected into a mixed slurry reactor at a flow rate of 2 L·min^–1^.

**Figure 1:**
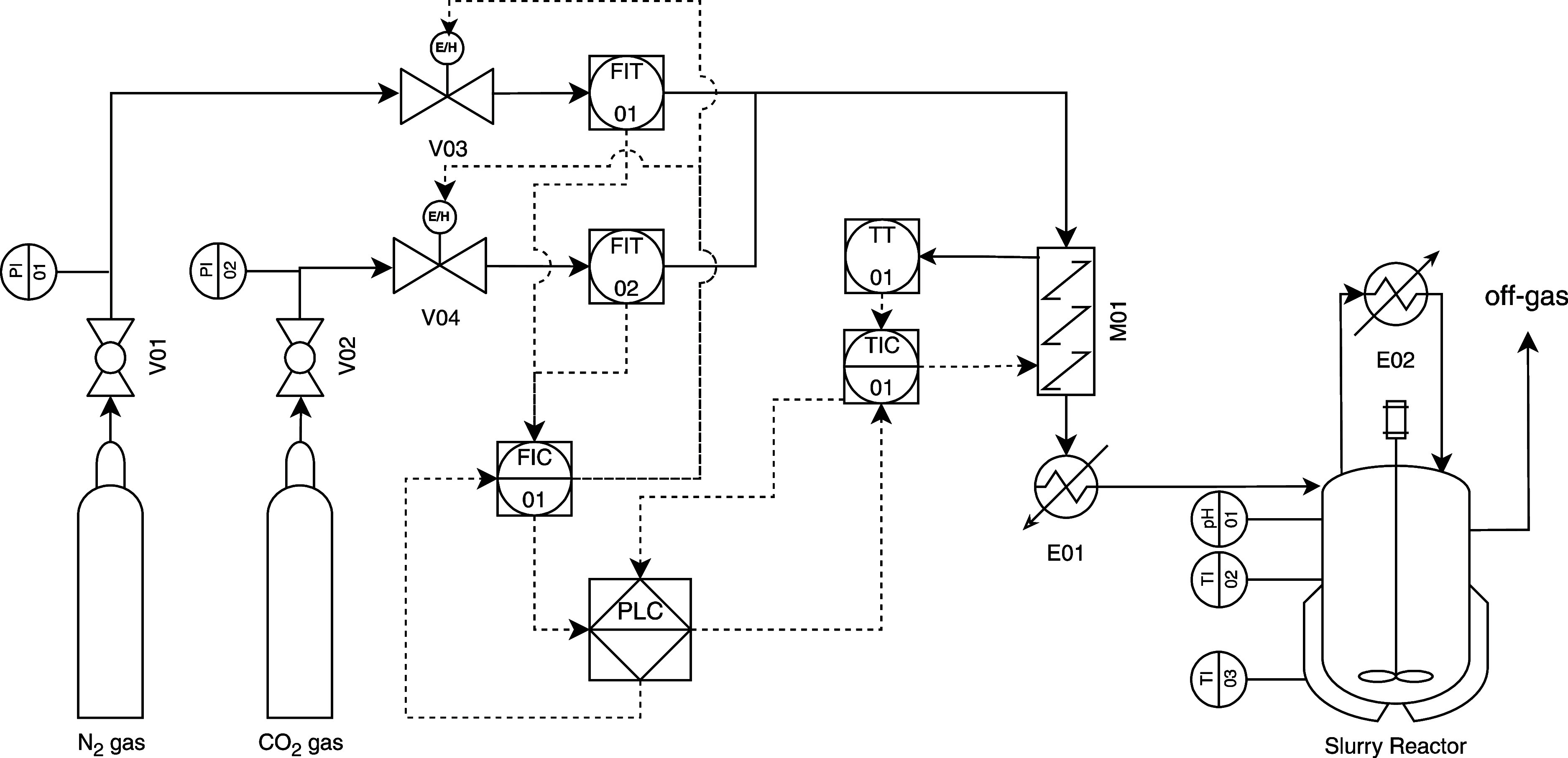
Schematic diagram of the wet semicontinuous carbonation setup.

Temperature was selected as the controlled variable because it exerts a dual influence on the process by affecting both CO_2_ solubility and reaction kinetics.^31^ Capturing sufficient temperature variation was necessary to enable the precise estimation of kinetic model parameters, particularly the activation energy. Pure water was used without additives that could influence ionic activity (e.g., salts^32^) or mass transfer (e.g., nanoparticles^33^), in order to avoid altering diffusion coefficients and to maintain the validity of the underlying modeling assumptions. The solid-to-liquid ratio was chosen based on values frequently reported as optimal in mineral carbonation literature.^27,28,31^ The gas flow rate was adjusted to ensure sufficient CO_2_ supply according to Henry’s law, while avoiding channelling, thereby preserving effective gas–liquid contact.^20^

The apparatus contains CO_2_ and N_2_ gas capsules equipped with ball valves (V01 and V02 in Figure 1) to direct gas to the control valves (V03 and V04 in Figure 1). These valves, triggered by a flow controller (FIC 01), operate after measuring the gas stream flows using FIT 01 and FIT 02. The user-controlled gas streams are then directed to a mixer and furnace (M01 and E01), regulated by TIC 01, to adjust the temperature to the reaction set point before being injected into the reactor. These controllers are part of a larger Distributed Control System (DCS), which allows for adjusting process variables via a Human–Machine Interface (HMI).

The reactor is equipped with a thermal water bath and thermometers for monitoring the bath and reactor temperatures during the reaction time to ensure the local uniformity and isothermal behavior through the batch process. This reactor is an agitated and bubbled system, fitted with a condenser (E02) to return the vapor stream, and a pH meter probe. It operates semicontinuously by holding the slurry while passing gas through and venting.

In each experiment, the slurry was prepared by dispersing the RCFs in water, stabilizing the mixture at the target batch temperature, and injecting the gas mixture at matching temperature conditions. After each experiment, cRCFs were collected and oven-dried at 105 °C for subsequent analysis.

### 2.3 Characterization Tests and Metrics for CO_2_ Mineralization

The chemical composition (oxides in % weight and normalized to 100%) of the material was obtained by X-ray fluorescence, PANalytical AXIOS. The quantitative crystalline and amorphous contents of the RCFs were determined by X-ray diffraction using an Empyrean diffractometer (Malvern-PANalytical) equipped with a multistrip PIXcel3D detector, Cu Kα radiation, and Bragg–Brentano optics. Measurements were conducted with an internal standard (20 wt % α-Al_2_O_3_) in the range of 5° to 120 2θ and sample spinning. Analyses of the samples were performed with the Rietveld method following the fundamental parameters approach implemented in TOPAS V4.2 (Bruker-AXS). Mass losses and CO_2_ content were quantified by thermogravimetric analysis (TGA) using a Jupiter STA 449 F3 (NETZSCH) with a heating rate of 10 °C/min from 25 to 1075 °C and a nitrogen gas flow (70 mL/min).

Density of RCFs and cRCFs were measured using a helium pycnometer AccuPyc II 1340. Particle morphology and geometry were investigated using a scanning electron microscope (SEM, Zeiss Supra VP55) with a field emission gun operating up to 25 kV accelerating voltage and 30 μm of aperture size. Elemental mapping of the indentation areas is performed using a Quantax Energy-Dispersive X-ray Spectroscopy (EDS) system (XFlash 5010 with 10 mm^2^ detection area and 127 eV fwhm/MnKα) coupled to the SEM. Attenuated Total Reflection (ATR) spectroscopy measurements were obtained on a Tensor II spectrometer (Bruker Optics, Ettlingen, Germany) with a deuterated triglycine sulfate (DTGS) detector and a golden gate ATR cell with diamond crystal (Specac LTD, Orpington, UK). Each spectrum is the average of 64 scans acquired over 2 min in the range 400–4000 cm^–2^ with a spectral resolution of 2 cm^–1^. The evaluation of the spectra was perfomed by OPUS (Bruker Optics). Particle size distributions were obtained with a Malvern Mastersizer 2000 with hydro 2000S using ethanol as dispersant. Specific surface area (SSA) was measured using a Quantachrome Nova 4000e (Quantachrome Instruments, Boynton Beach, FL, USA) with Nitrogen as probe and liquid nitrogen as coolant. All samples were dried under vacuum at 105 °C prior to analysis. Blended cements were obtained by mixing neat CEM I 52.5N with RCFs and cRCFs at replacement ratios of 5%, 10%, and 15%. Compressive strengths tests were conducted using EN mini prism with dimensions of 19 × 10 × 44 mm (width × height × length). Each mortar was composed of blended cement, sand, and water at a constant mass ratio set at 1:3:0.5 (% in relation to the mass of binder). At the given curing age, each blend was tested in triplicate.

The CO_2_ uptake, denoted as α, is a metric that indicates the net CO_2_ sequestration in the mass fraction of the RCFs during carbonation. It is evaluated at a set of discrete sampling times *t*_*s*_*p*__ ∈ {*t*_*s*_0__, *t*_*s*_1__, ..., *t*_*s*_N__ }, where *N* is the number of sampling points after starting the reaction, and *t*_*s*_0__ = 0 denotes the precarbonation baseline.

To isolate the CO_2_ fixed by carbonation, the uptake at time *t*_*s*_p__ is calculated by correcting for the CO_2_ content already present in the material at *t*_*s*_0__. It is calculated using eq 1, which is based on the gravimetric changes between 500 to 900 °C associated with CO_2_ content and the total mass loss up to 975 °C serving as normalization basis—as described in eqs 2 and ([Disp-formula eq3]), respectively. To note, outside the parentheses, the difference in CO_2_ is rescaled by the total mass loss of the RCFs ensuring that the results are meaningful and comparable to the starting feed of the process.

$$\alpha \left(t_{s_{p}}\right)=\left[\frac{\Delta m_{t_{s_{p}},500-900}}{100-\Delta m_{t_{s_{p}},30-975}}-\frac{\Delta m_{t_{s_{0}},500-900}}{100-\Delta m_{t_{s_{0}},30-975}}\right]\times \frac{100-\Delta m_{t_{s_{0}},30-975}}{100}$$ 1

where:

$$\Delta m_{t_{s_{p}},500-900}=m_{t_{s_{p}},500{}^{\circ}C}-m_{t_{s_{p}},900{}^{\circ}C},\mathrm{and}$$ 2

$$\Delta m_{t_{s_{p}},30-975}=m_{t_{s_{p}},30{}^{\circ}C}-m_{t_{s_{p}},975{}^{\circ}C}$$ 3

The carbonation efficiency, *X*(*t*_*s*_p__), see eq 4, is the metric that normalizes the CO_2_ uptake by considering the material theoretical carbonation potential. The theoretical potential, *Th*_CO_2__, is calculated using the modified Steinour equation, see eq 5, which uses the chemical composition from XRF as input.^34^

$$X\left(t_{s_{p}}\right)=\frac{\alpha \left(t_{s_{p}}\right)}{{\mathrm{Th}}_{{CO}_{2}}}$$ 4

$${\mathrm{Th}}_{{\mathrm{CO}}_{2}}=\frac{44}{56}\cdot \left(CaO-\frac{56}{100}\cdot \%{CaCO}_{3}-\frac{56}{80}\cdot SO_{3}\right)+1.091\cdot MgO$$ 5

### 2.4 Candidate Kinetic Models and Key Modifications

To describe the mineral carbonation process under wet conditions, different product layer diffusion-limited Shrinking Core Models were selected as candidate models. These models are reported to be more predictive and relevant to mineral carbonation processes when the reaction extends toward equilibrium and becomes limited due to mass transport through the product layer.^27,28^ The selected models are summarized in Table 1, including two models that are developed based on the approach proposed by Miao et al.^27^ to complement the geometric dependency of product layer growth regime by adding 1-D and 3-D growth systems in plate-shaped and spherical particles.

**Table 1:** Structure of Candidate Product Layer Diffusion Limited Shrinking Core Models

Model name	Remarks	Structure
BSCM_f,c_	D: *constant*	$k_{a}=\frac{16\cdot C_{0}\cdot D_{0}}{\rho \cdot {d_{p0}}^{2}},,\frac{dX}{dt}=-\frac{k_{a}}{\ln\left(1-X\right)}$
	Diffusion: *Fick*	
	Geometry: *cylindrical*	
BSCM_f,s_	D: *constant*	$k_{a}=\frac{24\cdot C_{0}\cdot D_{0}}{\rho \cdot {d_{p0}}^{2}},,\frac{dX}{dt}=\frac{k_{a}}{-2+2\cdot {\left(1-X\right)}^{-1/3}}$
	Diffusion: *Fick*	
	Geometry: *spherical*	
MSCM_f,p_	D: *dynamic*	$k_{a}=\frac{8\cdot M_{\mathrm{Ca}O}\cdot C_{0}\cdot D_{0}}{\rho \cdot f_{\mathrm{Ca}O}\cdot {d_{p0}}^{2}},,\frac{dX}{dt}=\frac{k_{a}\cdot \exp\left(-\theta \cdot k_{a}\cdot t\right)}{2\cdot X}$
	Diffusion: *Fick*	
	Geometry: *plate*	
MSCM_f,c_	D: *dynamic*	$k_{a}=\frac{8\cdot M_{\mathrm{Ca}O}\cdot C_{0}\cdot D_{0}}{\rho \cdot f_{\mathrm{Ca}O}\cdot {d_{p0}}^{2}},,\frac{dX}{dt}=-\frac{2\cdot k_{a}\cdot \exp\left(-\theta \cdot k_{a}\cdot t\right)}{\ln\left(1-X\right)}$
	Diffusion: *Fick*	
	Geometry: *cylindrical*	
MSCM_f,s_	D: *dynamic*	$k_{a}=\frac{8\cdot M_{\mathrm{Ca}O}\cdot C_{0}\cdot D_{0}}{\rho \cdot f_{\mathrm{Ca}O}\cdot {d_{p0}}^{2}},,\frac{dX}{dt}=-\frac{\frac{3}{2}\cdot k_{1}\cdot \exp\left(-\theta \cdot k_{a}\cdot t\right)}{\left({\left(1-X\right)}^{1/3}-1\right)}$
	Diffusion: *Fick*	
	Geometry: *spherical*	
MSCM_p,p_	D: *dynamic*	$k_{a}=\frac{4\cdot C_{0}\cdot D_{0}}{\rho_{{\mathrm{Ca}CO}_{3}}\cdot {d_{p0}}^{2}},,\frac{dX}{dt}=\frac{k_{a}\cdot \exp\left(-k_{a}\cdot \theta \cdot t\right)}{X}$
	Diffusion: *Parabolic*	
	Geometry: *plate*	
MSCM_p,c_	D: *dynamic*	$k_{a}=\frac{8\cdot C_{0}\cdot D_{0}}{\rho_{{\mathrm{Ca}CO}_{3}}\cdot {d_{p0}}^{2}},,\frac{dX}{dt}=\frac{k_{a}\cdot \exp\left(-\theta \cdot k_{a}\cdot t\right)}{\left({\left(1-X\right)}^{-0.5}-1\right)}$
	Diffusion: *Parabolic*	
	Geometry: *cylindrical*	
MSCM_p,s_	D: *dynamic*	$k_{a}=\frac{12\cdot C_{0}\cdot D_{0}}{\rho_{{\mathrm{Ca}CO}_{3}}\cdot d_{p0}^{2}},,\frac{dX}{dt}=\frac{k_{a}\cdot \exp\left(-k_{a}\cdot \theta \cdot t\right)}{\left(1-{\left(1-X\right)}^{1/3}\right)\cdot {\left(1-X\right)}^{-2/3}}$
	Diffusion: *spherical*	
	Geometry: *plate*	

Two diffusion-based Shrinking Core Models are formulated to address the one- and three-dimensional growth due to carbonation by leveraging the parabolic diffusion law, which is reported to have a better description of the diffusion through solid phases (product layer).^35^ The models are formulated based on the following assumptions:^27^

a. CO_2_ acts as an excess reactant, and the slurry remains saturated with it at all times.
b. The particle geometry and size stay constant throughout the reaction.
c. CO_2_ (aq) and mineral particles are evenly distributed within the slurry.
d. Temperature is uniform and remains constant during the reaction process.
e. CO_2_ diffuses molecularly, and chemical reactions are restricted to carbonation of CaO on the solid interface.
f. The effective diffusion coefficient changes over time, and the reaction is primarily diffusion-controlled.
g. The reaction follows the quasi-steady-state approximation.

The model derivation procedure is represented in Supporting Information, which results in a 1-D model, i.e., MSCM_p,p_ and a 3-D model, i.e., MSCM_p,s._ The structure of the two models is included in Table 1 together with other candidate particle-fluid Shrinking Core Models used to predict carbonation kinetics. It includes model name as abbreviation, description based on the diffusion law, intrinsic diffusion coefficient, particle geometry, and structure of the kinetic rate expression. The models are categorized as follows:

- Basic Shrinking Core models (BSCMs): Assuming a constant diffusion coefficient and a cylindrical (2-D) or spherical (3-D) particle geometries.
- Modified Shrinking Core models (MSCM): Accounting for the time-dependency of the diffusion coefficient and divided into:
  - Fickian diffusion-based models: Developed for plate, cylindrical, and spherical geometries.
  - Parabolic diffusion-based models: Developed for plate, cylindrical, and spherical geometries.

The model nomenclature follows a structural convention, where the prefix BSCM or MSCM identifies the model type. This prefix is followed by two subscripts: the first subscript denotes the diffusion mode (*f* for Fickian and *p* for parabolic), while the second subscript indicates the particle geometry (*p* for the plate, *c* for the cylinder, and *s* for the sphere).

These models consider dependency of apparent reaction coefficient (*k*_a_) on variables like: Dissolved CO_2_ concentration in water (*C*_0_), intrinsic diffusion coefficient through the product layer (*D*_0_), density of RCFs (ρ), initial diameter of particles (*d*_*p*0_), molar mass of CaO (*M*_CaO_), mass fraction of CaO in raw material available for carbonation (*f*_CaO_), and molar density of the reaction product (ρ_CaCO_3__). This arrangement lets the carbonation efficiency (*X*) be dependent only on time (*t*), apparent reaction coefficient (*k*_a_), and decay ratio (θ).

When applied to wet carbonation systems, these models require two key modifications to account for the concentration of diffusing CO_2_ and the system temperature dependency:

1. **Correlation of dissolved CO_2_ concentration (*C*_0_) with partial pressure (*P*_CO_2__):** The concentration of the diffusive species (CO_2_) must be correlated with its partial pressure in the injected gas. This modification accounts for the effective concentration of dissolved CO_2_ at the mineral particle surface by considering the liquid–gas equilibrium between the reactor liquid phase and the CO_2_-containing injected stream, which is treated as the excess reactant. Assuming the RCFs-containing liquid phase is saturated with CO_2_, Henry’s law can be applied to calculate the dissolved CO_2_ concentration in the aqueous (water) phase, denoted as *C*_0_. eq 6 is a temperature-dependent correlation for the CO_2_-water equilibrium and reads as follows:
$$C_{0}=P_{{CO}_{2}}\cdot 35\cdot \exp\left[2400\cdot \left(\frac{1}{T}-\frac{1}{T_{\mathrm{ref}}}\right)\right]$$ 6
2. **Decomposition of the intrinsic diffusion coefficient (** ***D***_**0**_ **):** The second modification introduces temperature dependency in the reaction kinetics by decomposing the intrinsic diffusion coefficient (*D*_0_) rather than the apparent reaction coefficient (*k*_a_). In this approach, *D*_0_ is expressed as the product of the pre-exponential factor (*A*) and an exponential term involving the activation energy (*E*_a_) according to the Arrhenius eq (eq 7). This decomposition incorporates thermal dependency into the model structure, improving its predictive capability for systems where temperature varies.^36^
$$D_{0}=A\cdot \exp\left(\frac{-E_{a}}{R\cdot T}\right)$$ 7

### 2.5 Model Identification Approach

A systematic approach was employed for model identification, targeting two main objectives: discrimination between model candidates and the calibration of the selected model. Figure 2 illustrates this approach, which comprises four main steps: (1) Preliminary calibration, (2) Estimability analysis, (3) Calibration and discrimination, and (4) Validation. Each of these steps is described in more detail as follows.

1. **Preliminary calibration:** This initialization step aims at estimating the parameters of all model candidates and at quantifying the associated modeling uncertainty. It is formulated as a nonlinear regression problem, employing the Least Squares (eq S1) as the loss function and *R* ^2^ (eq S2) as a metric for model predictability.^37^ To evaluate the precision of the estimation, a *t* test is applied by calculating the *t*-value for each parameter (eq S5) and comparing it against the reference *t*-values. Higher *t*-values indicate greater precision in parameter estimation.^38^
2. **Estimability analysis:** Following the preliminary calibration of model candidates, parameters are ranked based on their impact on model responses employing Estimability Analysis (EA) with Orthogonalisation.^39^ EA-Orthogonalisation removes dependencies among parameters to ensure their independent contribution. It ranks parameters based on significance, calculates the corrected critical ratio, and selects the most important ones from the ranked list, prioritizing those with the lowest corrected critical ratio.
3. **Calibration and discrimination:** Once the models are analyzed and insignificant parameters fixed, the retained parameters are re-estimated, and their precision is re-evaluated using the same criteria described in Step 1. Models with parameters exhibiting low t-values are excluded from further consideration, because they cannot be calibrated with available experimental data set. Model discrimination is then conducted by calculating *P*-values (eq S4), which serve as a metric for the likelihood that a given model is the best descriptor of the system.^40^ In cases where *P*-values are similar—indicating comparable predictive behavior among models—the model with the fewest fixed parameters was selected.
4. **Validation**: Once a representative model with acceptable precision is identified, a validation test is conducted. The test follows the leave-one-out (LOO) method,^41^ where one entire reaction isotherm is iteratively removed, followed model recalibration using the remaining data, and by validating it against the excluded data. This iterative approach assesses the model robustness and ensures its reliability when exposed to unseen data.

**Figure 2:**
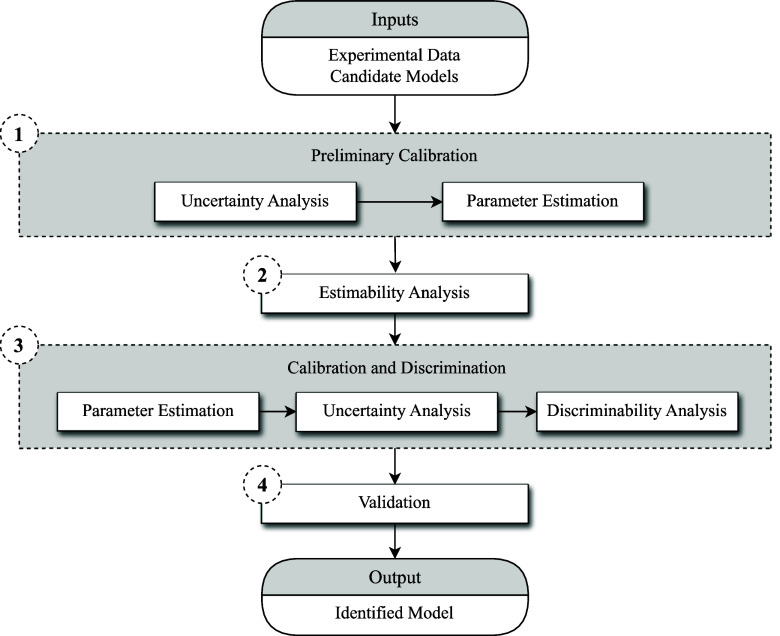
Schematic diagram of the model identification approach with the following steps: (1) Preliminary Calibration, (2) Estimability Analysis, (3) Calibration and Discrimination, and (4) Validation.

All of the aforementioned numerical procedures were carried out using the Python library MIDDoE.^42^

### 2.6 Model Sensitivity Analysis for Inputs

The contribution of process controls was evaluated using Global Sensitivity Analysis (GSA) with Sobol’s method.^43^ GSA-Sobol captures nonlinear interactions among process controls by computing the total Sobol index, which serves as a metric for their influence on carbonation efficiency over reaction time for the identified model. This method is applied to rank the impact of process controls.

## 3 Results and Discussion

### 3.1 Characterization of RCFs and cRCFs

Table 2 lists the chemical and mineral compositions of the RCFs employed in the study. SiO_2_ and CaO constitute the highest proportion of the sample, corresponding with the presence of quartz, calcite, and C–S–H (Ca_*x*_SiH_*y*_O_(*x*+2+*y*/2)_), the latter represented in the amorphous content.

**Table 2:** Chemical and Mineralogical Compositions of the Studied RCFs

Chemical composition		Mineralogical composition	
XRF	wt %	XRD	wt %
SiO_2_	50.96	Amorphous	42.4
Al_2_O_3_	5.61	Quartz	21.3
Fe_2_O_3_	2.41	Calcite (CaCO_3_)	19.1
CaO	36.06	Feldspars (Na, K, Ca)^c^	5.5
MgO	0.89	Clinker phases^d^	1.1
SO_3_	1.27	Portlandite (Ca(OH)_2_)	2.1
K_2_O	1.36	Other hydrated phases^e^	6.3
Na_2_O	0.80	Other minerals^f^	2.2
Others^a^	0.64		
LOI^b^, 975 °C	21.67		

TGA	wt %
Δ*m*_*t*_*s*0_,500–900_	10.78
Δ*m*_*t*_*s*0_,30–975_	19.76

a Mn_2_O_3_, TiO_2_, P_2_O_5_, SrO.

b Loss on ignition.

c Microcline, Labradorite, Lazurite.

d C_3_S, C_2_S, C_3_A.

e Ettringite, Monocarboaluminate, Hydrogarnet.

f Biotite, Kaolinite, Cordierite.

XRD results (Figure S3) reveal the presence of clinker minerals such as C_3_S (3CaO·SiO_2_), C_2_S (2CaO·SiO_2_), and C_3_A (3CaO·Al_2_O_3_), which is likely due to the coarser grinding practices typical of past decades, in contrast to the higher fineness targeted in more recent years. Among the hydrated cement phases, ettringite (3CaO·Al_2_O_3_·3CaSO_4_·32H_2_O), monocarboaluminate (3CaO·Al_2_O_3_·CaCO_3_·11H_2_O), portlandite (Ca­(OH)_2_), and hydrogarnet (3CaO·(Al_2_O_3_)_0.8_(Fe_2_O_3_)_1.2_·0.84SiO_2_·4.32H_2_O) were identified. Additionally, numerous mineral phases, including alkali-feldspars, biotite, kaolinite, and cordierite, were also found. These latter minerals are associated with sand and aggregates, highlighting the challenge of addressing an industrial material.

For reliable quantification using the Rietveld method, the optimal structure files of the phases were verified and cross-checked with the mass composition. Mass loss between 500 to 900 °C and between 30 to 975 °C is also included in Table 2, as these values serve to define inherited natural carbonation of RCFs and, as the basis of normalization calculations as carbonation progresses.

The CaCO_3_ content in the material prior to carbonation was estimated from TGA results as 10.78/0.44 = 24.5%, indicating that nearly 48% of the CaO is already carbonated, thereof the mass fraction of CaO available for carbonation (*f*_CaO_) is estimated around 14.6%. Mass balance calculations further suggested the presence of approximately 23% C–S–H and 8.6% hydrogarnet.

After carbonation, combined XRD and mass balance analyses indicate a significant increase in the relative amount of CaCO_3_ and the total consumption of portlandite; the content of clinker phases decreases while feldspars and other minerals remain roughly constant.

Figure 3 illustrates the particle size distribution of the RCFs and two carbonated samples at 65 °C after 10 and 180 min (see Figure S4 for the full distribution set). The sieving (pretreatment) on the fines resulted in RCFs with a narrow size distribution, which progressively shifts toward finer particles as carbonation progresses. For instance, at start 10% of RCF particles were smaller than 45 μm, while upon carbonation at 65 °C this fraction increased to 38.4% after 10 min and further rose to 64% after 180 min of reaction.

**Figure 3:**
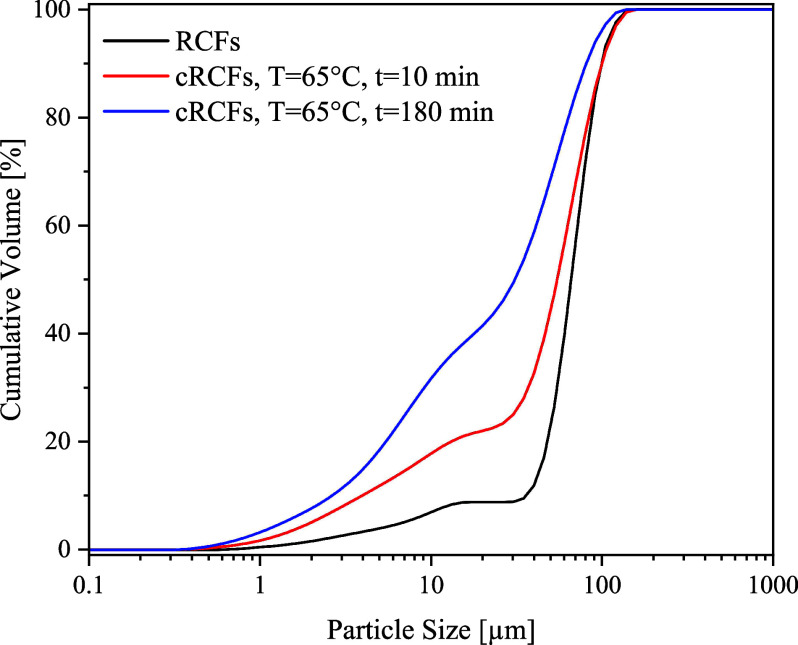
Particle size distribution for the RCFs and cRCFs at 65 °C after 10 and 180 min of reaction.

Figure 4 displays SEM images of the industrial RCFs (a–c) and of the carbonated counterpart at 65 °C for 180 min (d–g). Results under this condition are presented as no significant variations in the shape or size of the particles were observed at any of the evaluated temperatures based on SEM images. The numbered circles mark the location where EDS point spectra were acquired (1–4). The RCFs consist of irregular, angular particles with rough surfaces (Figure 4a–c). Two representative surface types are apparent in (Figure 4b): a relatively smooth, blocky particle (point 1) and a particle coated by a microcrystalline layer (point 2). EDS of point 1 is consistent with a feldspar-rich particle, whereas the coating at point 2 likely contains C–S–H mixed with a minor content of carbonate-bearing mineral. A definitive phase assignment cannot be made from EDS alone, because carbonate may occur either as CaCO_3_ or as carboaluminate phases, as previously presented by the XRD results above.

**Figure 4:**
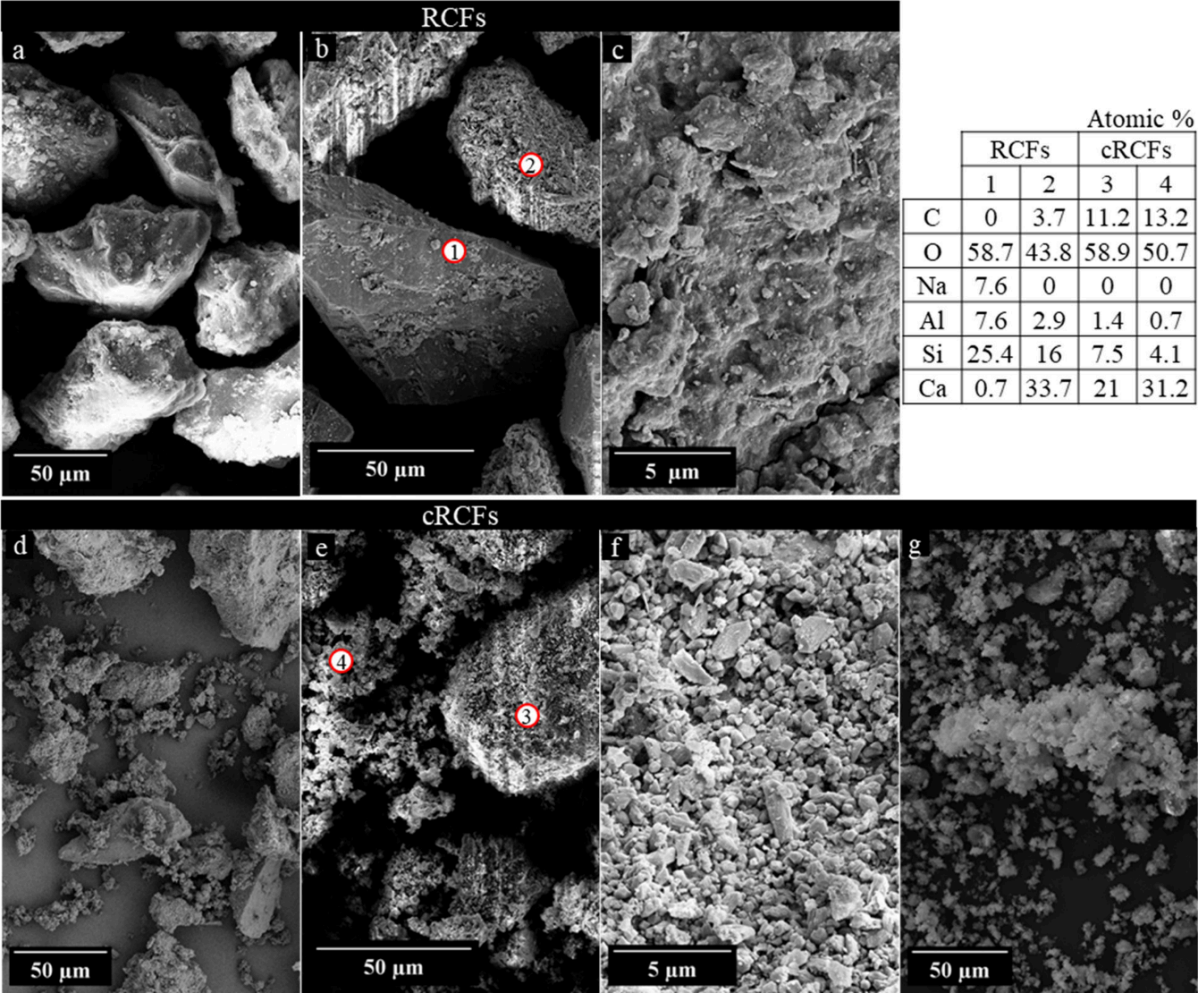
SEM images of (a–c) RCFs, (d–f) cRCFs, and (g) cRCFs < 22 μm at 65 °C, after 180 min. Red circles indicate location of EDS point analysis (1–4).

After carbonation (Figure 4d–f), the coarse particles are covered by abundant fine polyhedral (rhombohedral-like) microcrystals. The fine particles form a discontinuous surface layer, at least partially composed of CaCO_3_ as indicated by increased C in EDS results at points 3–4, consistent with XRD/TGA. Additionally, decreased Si and Al relative to Ca also suggest the presence of decalcified C–S–H and Si­(Al)-gel among the carbonation products.

Sieving the carbonated sample through a 22 μm mesh isolates a fine fraction composed of agglomerates of plate-like particles (Figure 4g).TGA measurements (Figure S5) confirmed that this finer fraction contains approximately 70% CaCO_3_. This observation is particularly interesting, as particles smaller than 45 μm had been mostly removed from the original RCFs prior to carbonation. This suggests that the conditions provided by wet carbonation—e.g., turbulences by stirring and CO_2_ flow—favor the detachment and agglomeration of CaCO_3_ formed on the particle surface.^44^

Regarding the formation of calcium carbonate, the higher proportion of these minerals observed on the surface indicates that the crystals do not grow in a confined space but rather result from a transport-limited process. This ultimately passivates the surface and slows down the reaction, as reported elsewhere.^16,30,45^

Regarding the specific surface area (BET), cRCFs have a value of 18.0 m^2^/g, which is nearly four times higher than that of the RCFs at 4.7 m^2^/g, reflecting significant microstructural changes induced by carbonation. The increase in surface area may result from the disaggregation of inert particles caused by the carbonation of the binder. Note that the carbonate fines formed below 22 μm exhibit an even greater specific surface area 22.6 m^2^/g, yet this fraction only accounts for about 20 wt % of the carbonated product. This suggests that the remaining 80 wt % also experienced a considerable increase in surface area, likely attributed to the carbonation of C–S–H and formation of the Si­(Al)-gel, which is known to have a specific surface area of approximately 100 m^2^/g and to contain more loosely bound water.^18,46^

ATR spectra in Figure S6 indicate the formation of a Si­(Al)-gel, as there is a shift of the asymmetric stretching vibration ν_3_Si–O of C–S–H from 967 to higher wavenumbers in the range of 1025–1032.^47,48^ This shift is consistent with increased silicate polymerization and approaches the band position of amorphous SiO_2_ (Aerosil). For comparison, a C–S–H with Ca/Si = 5/6 (Calcium to silica ratio) is included as a reference to infer the calcium content in the RCFs. The spectra also display bands attributable to carbonate stretching (ν_4_C–O ∼ 713 cm^–1^, ν_2_C–O ∼ 873 cm^–1^, ν_3_C–O ∼ 1390–1405 cm^–1^).^49^ Notably, the spectra are very similar for all four temperatures tested, consistent with the SEM description presented above and the carbonation efficiency, as discussed in section 3.2. Furthermore, the observed increase in density from 2.46 g/cm^3^ to 2.57 g/cm^3^ after carbonation is consistent with the formation of CaCO_3._ ^50^

Figure 5 presents the compressive strength of blended cement mortars prepared by using RCFs and cRCFs (carbonated at 25 °C) at replacement ratios of 5%, 10%, and 15%. The results suggest that up to 10% substitution with cRCFs is feasible without significantly compromising the mechanical performance of the blended cement. In terms of the influence of carbonation on strength development, both early and late-age responses are comparable between RCFs and cRCFs samples, although a slight improvement is observed for cRCFs at early ages. Previous studies have attributed such improvements to the presence of Si­(Al)-gel and the increase in specific surface area, which enhance the reactivity and rapid synergy between cRCFs and cement.^18,51^

**Figure 5:**
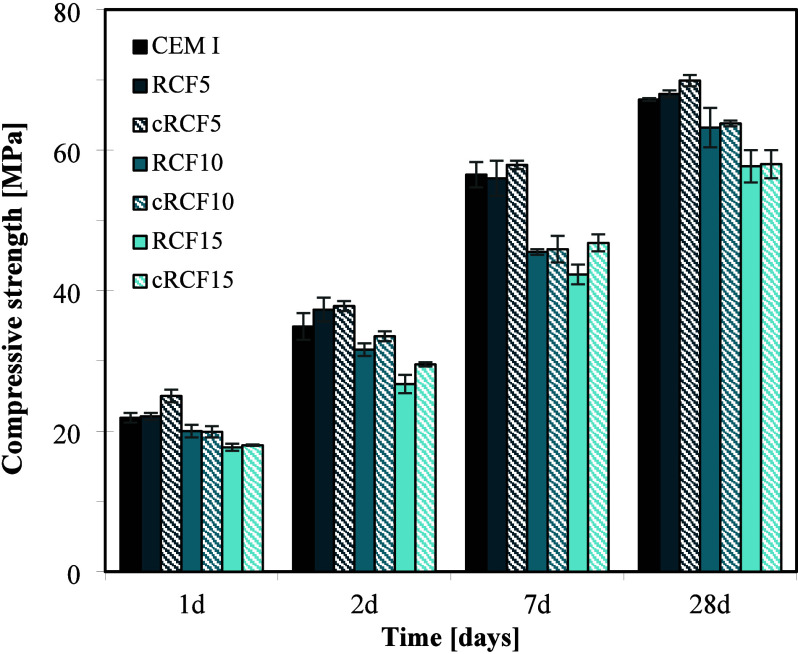
Compressive strength of blended cement mortars prepared with 5, 10, and 15% RCFs and cRCFs at 25 °C as partial replacements of CEM I.

However, it is important to note that most of the positive outcomes reported in the literature involve either the carbonation of pure cement paste or synthetic RCFs with a higher content of carbonatable phases, which provide a greater source of reactivity. In contrast, the RCFs used in this study exhibit a more limited availability of carbonatable materials, which constrains their overall performance enhancement.

### 3.2 Carbonation Efficiency

Figure 6a illustrates the carbonation efficiency used as a metric to evaluate the reaction progress of wet carbonated samples over time. Carbonation efficiency after 180 min of reaction ranges from 0.74 to 0.81, corresponding to a CO_2_ uptake between 87 and 95 kg per tonne of RCFs. As a side note, the final carbonation might also include natural carbonation occurring during service life. Therefore, considering only the CaO content in the RCFs, the total CO_2_ uptake is estimated at 199 kg per tonne of RCFs, with an overall efficiency of 90%. Limited to the tested material and carbonation conditions, no significant changes were observed throughout the analyzed period, with carbonation efficiency variations remaining below 0.029 across all tested conditions.

**Figure 6:**
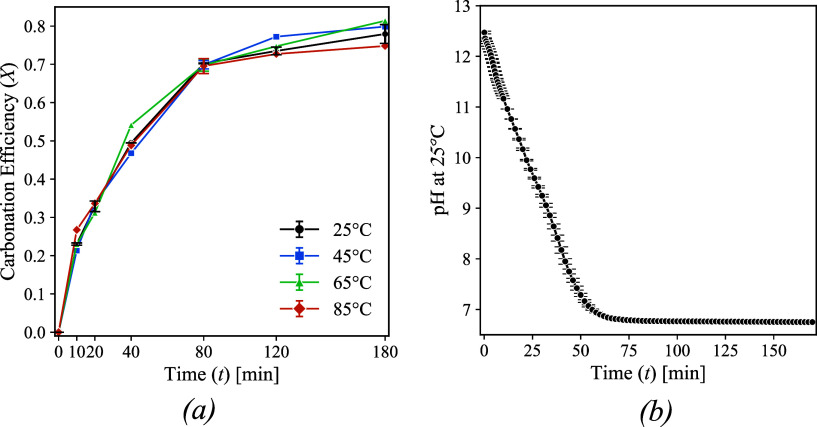
Measurements of (a) carbonation efficiency of the RCFs at temperatures of 25, 45, 65, and 85 °C and (b) pH at 25 °C during reaction time.

To ensure confidence in the reproducibility of the data, the experiment at 25 °C was repeated. The replication yielded a negligible average standard deviation of 0.01 for pH and carbonation efficiency measurements, reinforcing the reliability of the observed trends.

Figure 6b presents the pH values recorded during the experiments. Higher pH values were observed at the onset of the reaction, likely due to the dissolution of CaO from Ca­(OH)_2_ during the initial minutes. Since the RCFs contained only a limited amount of Ca­(OH)_2_, this phase was fully consumed within the first 10 min of carbonation (Figure S5). Consequently, the subsequent carbonation of the RCFs was primarily driven by the decalcification of C–S–H.

Interestingly, despite temperature variations, the overall extent of carbonation remained similar across all of the tested conditions. This outcome suggests a counterbalancing effect, wherein the influence of temperature on the diffusion coefficient (which according to model formulations defines the rate of the reaction) and CO_2_ dissolution offset each other. This aspect is further discussed in section 3.3.2.

### 3.3 RCFs Carbonation Kinetic Modeling

In this section, two kinetic models are formulated based on Shrinking Core Models and the parabolic diffusion law in product layer – for cases where diffusivity coefficient changes occur. Using the set of rival models from Table 1, the proposed workflow for model screening, discrimination, calibration, and validation (Figure 2) is applied, alongside the experimental data from RCFs wet carbonation.

#### 3.3.1 Step 1: Preliminary Calibration of Models

Table 3 presents the preliminary estimates for parameters (Figure 2, Step 1) of different models before any screening is applied to models. The 95% *t*-values of these estimations are also calculated and compared with the reference *t*-value of this system, which is 2.04. It is observed that for all models estimations of *A* are not precise enough, as indicated by lower *t*-values. Additionally, the decay ratio θ was not estimated with an acceptable precision for model MSCM_p,c_. While all models demonstrate acceptable predictive performance, MSCM_f,p_ and MSCM_p,p_ are the most predictive, exhibiting the same *R* ^2^ value.

**Table 3:** Parameter Estimates and *t*-values for the RCFs Carbonation Candidate Models

	*A*		*E*_a_		θ		
Models	Estimation [m^2^·s^–1^]	*t*-Value	Estimation [J mol^–1^]	*t*-Value	Estimation	*t*-Value	*R* ^2^
BSCM_f, c_	6.0938 × 10^–07^	1.852	20211	14.075	-	-	0.9722
BSCM_f, s_	3.0709 × 10^–07^	1.905	20209	14.459	-	-	0.9774
MSCM_f, p_	3.1837 × 10^–07^	1.904	20253	14.468	1.2140	12.086	0.9841
MSCM_f, c_	8.7542 × 10^–08^	1.808	20216	13.734	2.1995	4.406	0.9814
MSCM_f, s_	3.7633 × 10^–08^	1.837	20222	13.936	6.9512	6.716	0.9823
MSCM_p, p_	1.3414 × 10^–06^	1.904	20252	14.467	2.4279	12.084	0.9841
MSCM_p, c_	3.8531 × 10^–07^	1.780	20197	13.515	0.8469	1.475	0.9802
MSCM_p, s_	1.8455 × 10^–07^	1.707	20187	12.941	1.8627 × 10^–07^	13.936	0.9787

#### 3.3.2 Step 2: Estimability Analysis of Models

To select the parameters that can be estimated based on the available experimental data set, estimability analysis was carried out. Table 4 (derived from the corrected critical ratios in Figure S7) presents the results of the estimability analysis for parameter ranking and subset selection. Across all models, *E*_a_ is identified as the most appropriate parameter to retain and pre-exponential factor as the most insignificant.

**Table 4:** Estimability Analysis Results for Different Candidate Models

	*A*		*E*_*a*_		θ	
Models	Rank	Selected	Rank	Selected	Rank	Selected
BSCM_f,c_	2	no	1	yes	—	—
BSCM_f,s_	2	no	1	yes	—	—
MSCM_f,p_	3	no	1	yes	2	no
MSCM_f,c_	3	no	1	yes	2	no
MSCM_f,s_	3	no	1	yes	2	no
MSCM_p,p_	3	no	1	yes	2	yes
MSCM_p,c_	2	no	1	yes	3	no
MSCM_p,s_	3	no	1	yes	2	no

Interestingly, although the experimental data indicated a limited direct influence of temperature on observable outcomes, still *E*_a_—the parameter most strongly tied to temperature dependence—is identified as the dominant contributor. This implies that, while temperature may not appear to affect the process significantly, the underlying kinetics must compensate for temperature-related effects such as those arising from Henry’s law. This is a key insight that illustrates how the model can validate mechanistic assumptions even when direct trends are not evident in the measurements.

The persistent insignificance of the pre-exponential factor can be attributed to the well-known high correlation between *E*_a_ and *A* in Arrhenius-type formulations, eq 7, particularly within the narrow temperature range considered in this study.^52^

In the case of MSCM_f,p_ and MSCM_p,p_—identified as the most predictive models—this method suggests retaining *E*_a_ for both models, along with θ only for the formulated model, MSCM_p,p_, while fixing the remaining parameters. For the remaining models, only *E*_a_ is retained as a parameter to be estimated.

#### 3.3.3 Step 3: Model Calibration and Discrimination

Another round of parameter estimation and uncertainty analysis (Figure 2, Step 3) was conducted to reestimate only the selected parameters. Table 5 presents the variations in the *t*-values and calculated *P*-values for all identified models. These results suggest that MSCM_f,p_ and MSCM_p,p_ best represent the carbonation system. However, further discrimination between these models is challenging since the P-value for MSCM_p,p_ is only slightly higher than that of MSCM_f,p_. Given this marginal difference, additional criteria were considered to guide model selection. Notably, MSCM_p,p_ emerged as the stronger candidate as it also enabled a satisfactory estimation of the decay ratio (θ) which plays a key role in reaction termination and in determining the final equilibrium state of the reaction.

**Table 5:** *t*-Values of the Parameter Estimates and *P*-Values for RCFs Carbonation Candidate Models (after Fixing Parameters)

Models	*E*_a_ (*t*-value)	θ (*t*-value)	*P*-value
BSCM_f,c_	202.598	-	9.8083
BSCM_f,s_	208.033	-	12.2518
MSCM_f,p_	212.44	-	16.2518
MSCM_f,c_	201.537	-	12.1775
MSCM_f,s_	204.465	-	13.2978
MSCM_p,p_	133.650	12.3087	16.2525
MSCM_p,c_	197.996	-	10.8931
MSCM_p,s_	189.606	-	9.0672

For these reasons, MSCM_p,p_ is chosen over the other models and is further examined in terms of validation and behavior.

After fixing the pre-exponential factor, the estimated values of the other parameters remained unchanged, indicating that the optimal solution had already been reached; however, the precision of the estimates increased significantly. The effect of the increase in the precision of the estimates is illustrated in Figure 7 for the representative model.

**Figure 7:**
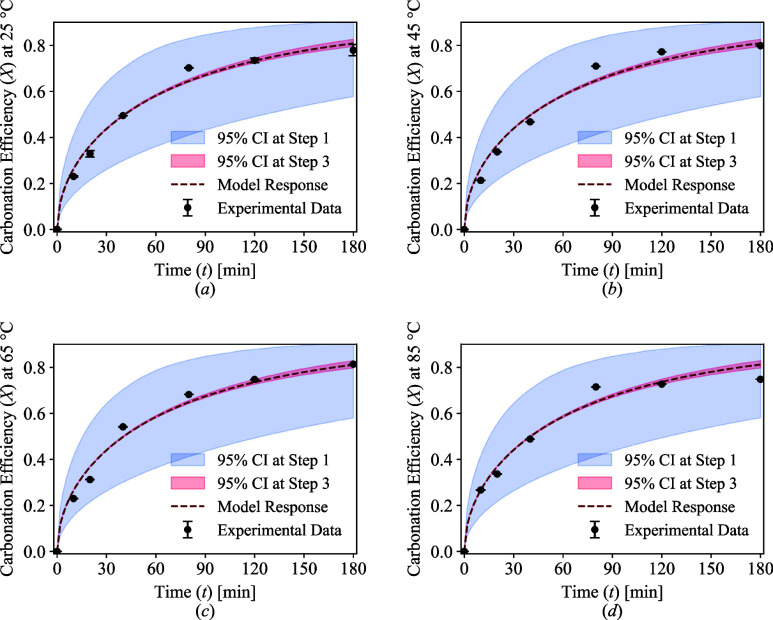
Experimental data and representative model response with 95% CI for the original model at Step 1 and the model at Step 3 of the RCFs after fixing pre-exponential factor for: (a) 25 °C, (b) 45 °C, (c) 65 °C, and (d) 85 °C. The colored area represents the impact of parametric uncertainty on model predictions.

#### 3.3.4 Step 4: Model Validation

The validation test (Figure 2, Step 4) confirmed the validity of the identified model, MSCM_p,p_, yielding a high validation *R* ^2^ of 0.9817 (±0.0040). This value is close to the calibration *R* ^2^ of 0.9841, demonstrating the model robustness and predictive reliability.

The aforementioned approach led to the conclusion that wet carbonation kinetics of RCFs can be predicted using a Modified Shrinking Core Model developed by considering diffusion through the product layer as the controlling step, with a time-variant effective diffusion coefficient and a parabolic diffusion law in a 1-D product growth mechanism. The pre-exponential factor, activation energy, and decay ratio for this model are estimated to be 1.34 × 10^–06^ m^2^·s^–1^, 20252 ± 0.01 J·mol^–1^ and 2.42 ± 0.18, respectively, with the error representing the 95% confidence interval. No error was attributed to the pre-exponential factor since its value was assigned during the final estimation task.

### 3.4 Mechanistic Insights through Comparison with RCP Carbonation

With the model performance within the explored design space, the physical meaning of the parameters is discussed by linking them to experimental measurements and comparing them with a model for a similar carbonation process. To this end, a kinetic model identified using our approach (Figure 2) was applied to published data on the carbonation of RCP,[Fn fn1] featuring a mean particle diameter of 8 μm and pure CO_2_ injection in a wet stirred reactor.^30^ Detailed results from each step of the algorithm are provided in the Supporting Information.

The identified model for RCP wet carbonation is a 2D growth model with a parabolic diffusion law and particles with cylindrical approximated geometry (MSCM_p,c_). This model belongs to the same group of modified Shrinking Core Models identified for RCFs wet carbonation (Table 1). The model was identified with a pre-exponential factor of 1.48 × 10^–8^ m^2^.s^–1^, activation energy 24191 J·mol^–1^, and decay ratio of 4.12; these are comparable to the ones obtained for the RCFs carbonation model.

The predictability of the RCP carbonation model for this data set indicates better performance of 2D and 3D growth mechanisms compared to 1D, as shown in Table S2. This could be attributed to the smaller particle size and the inherently homogeneous composition of the RCP, resulting in uniformly carbonated particles consisting of a mixed distribution of Si­(Al)-gel and CaCO_3_. The Si­(Al) gel is primarily responsible for the gel and capillary porosity.^30^ Conversely, for RCFs carbonation, the model demonstrates better predictive accuracy for a 1D growth mechanism rather than higher-dimensional growth, as evidenced by Table 4. This is likely because RCF particles are a composite of inert materials and hydrated cement paste. As a result, carbonation occurs primarily on the cement rich surfaces, leading to a more surface-oriented process. SEM observations show that RCP forms a compact and dense carbonation layer,^30^ whereas RCFs exhibit a one-sided carbonation layer composed of loosely packed, plate-shaped fine particles, as depicted in Figure 4.

The variation of the effective diffusion coefficient (*D*) through the product layer during the reaction was calculated for both materials and is presented in Figure 8 (the corresponding CO_2_ solubility at the end of the reaction is reported in Table S4 for RCFs). The results indicate a significant drop in the effective diffusion coefficient as the reaction progresses for both material but markedly for RCP carbonation. This is attributed to the higher reaction driving force, influenced by factors such as a higher CO_2_ partial pressure, higher CaO content, and finer particle size, which collectively enhance solubility and gas reactant availability. Despite the higher overall mass transfer rate in RCP, it is also clear from Figure 8 that carbonation of RCP occurs with a lower effective diffusion coefficient compared to that of RCFs carbonation. This is likely due to the formation of the Si­(Al) gel coating layer, which acts as a stronger barrier to diffusion. The results also demonstrate a high magnitude of change in the effective diffusion coefficient across different reaction temperatures. Higher temperatures result in increased diffusion rates, but this must be considered alongside CO_2_ solubility, which decreases as the temperature increases.

**Figure 8:**
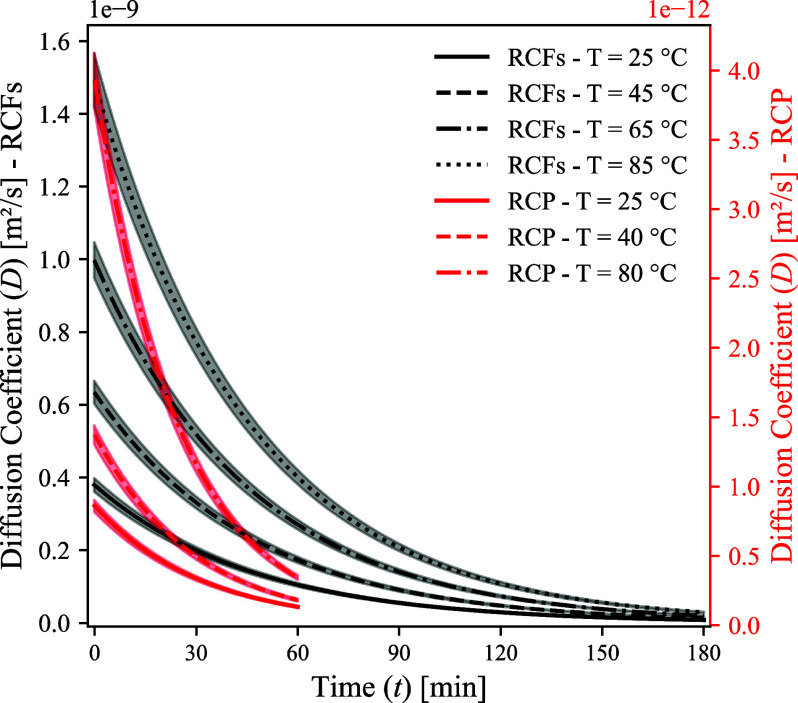
Variation of the effective diffusion coefficient for RCFs and RCP during wet carbonation at the investigated temperatures.

In the end, the material carbonation efficiency is a trade-off between increasing effect of temperature on species diffusion through the product layer or surface reaction (in case it becomes a limiting step) and the decreasing effect of it on solubility of CO_2_ in the liquid phase. The latter was estimated to be reduced to approximately one-fourth of its initial value when the temperature increased from 20 to 80 °C, as a consequence of vapor–liquid equilibrium conditions.

More importantly, as carbonation progresses, the effective diffusion coefficient becomes less sensitive to temperature variations regardless of the reaction temperature. Overall, temperature can increase the effective diffusion coefficient by a factor of 3–4 when raised from 25 to 80 °C; however, the decline caused by diffusion limitations in the product layer can lead to a reduction of up to 1 order of magnitude. Toward the end of the reaction, the decay ratio becomes the dominant factor rather than activation energy, which influences the temperature dependence of the process.

Finally, the overall impact of controllable input variables on the identified models for RCFs and RCP wet carbonation is assessed, since it can guide the selection of impactful process variables for the design of experiments—particularly with the aim of narrowing the feasible region toward an optimal zone of carbonation efficiency. To this end, the GSA-Sobol method was employed once again (Figure 2, Step 2); this time it was used to explore the design space defined by reaction temperature, CO_2_ partial pressure, and average particle size. Figure 9 illustrates the influence of these variables against reaction time (180 min), while keeping the model parameters fixed at their estimated values.

**Figure 9:**
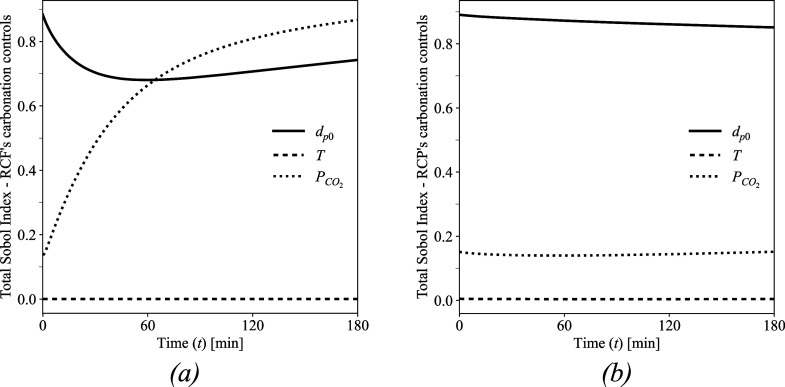
Total Sobol indices, which quantify the contribution of input variables to output variance, for controllable inputs in wet carbonation models identified for (a) RCFs and (b) RCP.

This analysis yields noteworthy insights. For instance, the temperature exhibits an almost negligible effect on carbonation performance for both materials. In contrast, particle size demonstrates a pronounced influence: it is the dominant factor for RCP carbonation throughout the reaction and plays a critical role in the early stages of RCFs carbonation, although its significance diminishes as the reaction approaches equilibrium. Note that the average particle size, while initially a controllable variable, is typically not adjustable during the process, i.e. it should be treated as a constant average over the course of the reaction.

As for the partial pressure of injected CO_2_, its effect is overshadowed by the dominant effect of the particle size in the case of RCP carbonation. However, in RCFs carbonation, its significance increases notably as the system approaches equilibrium. The greater influence of *d*_*p*0_ and *P*_CO_2__ compared to temperature further underscores their direct impact on the physical properties of the product layer, such as density, capillary or gel porosity, and pore size distribution. This highlights that implementing a progressively increasing pressure profile during the process, along with the use of smaller particle diameters, could enhance the carbonation efficiency.

## 4 Conclusions

The wet carbonation kinetics of RCFs was studied across 25–85 °C, reaching a maximum carbonation efficiency of 0.81, which corresponds to 95 kg CO_2_ per tonne of RCFs. Carbonated RCFs demonstrated an acceptable compressive strength development when incorporated at up to 10% in blended cement. This study showed a similar time progression for all the isotherms, a rarely reported behavior for mineral carbonation. A mechanistic kinetic model was developed and validated with a satisfactory predictive capability in this low-information system to explain this observation. The model, formulated as a one-dimensional parabolic-law diffusion Shrinking Core showed that the offset between diffusion coefficient and CO_2_ solubility changes, results in temperature independency of the process.

Deeper analysis using the same modeling approach on RCP carbonation revealed similar diffusion-limited behavior. However, RCFs formed a relatively loose product layer, underscoring the influence of particle shape, composition, and CO_2_ flow rate on diffusion resistance. The analysis also showed the greater importance of the average particle size in RCP carbonation and its time-dependent interplay with the CO_2_ partial pressure in RCFs carbonation.

Overall, this study demonstrates that a generalized Shrinking Core Model, supported by a systematic model identification strategy, can describe the diffusion-controlled carbonation of RCF particles, providing a basis for scaling up CO_2_ mineralization in cement-based wastes and process optimization. However, the model evaluation focused on temperature variability, while other influential factors—such as solid-to-liquid ratio, and gas velocity which affect mixing and species dissolution—were held constant. Accounting for these variables requires a more comprehensive, extra-particle modeling approach. Additionally, particle size distribution emerged as a key factor in sensitivity analyses, highlighting the need for further experimental work to confirm the model applicability across a range of particle sizes while addressing their distribution instead of an average representative particle size.

## Supplementary Material



## Footnotes

a RCP kinetic data were obtained by digitizing plots from ref 30.

## Notes

Formulation of models, model identification approach, model identification of RCP carbonation, supplementary characterization results (XRD patterns, PSD curves, TGA results, ATR spectra), estimability analysis results, and CO_2_ solubility data (PDF)

## List of Symbols

**Acronyms**
ATR: Attenuated Total Reflection
BSCMs: Basic Shrinking Core Models
CCUS: Carbon Capture, Storage, and Utilization
CI: Confidence Interval
cRCFs: Carbonated Recycled Concrete Fines
cSCMs: Carbonated Supplementary Cementitious Materials
DCS: Distributed Control System
EA: Estimability Analysis
EDS: Energy-Dispersive X-ray Spectroscopy
GSA: Global Sensitivity Analysis
HMI: Human–Machine Interface
LOO: Leave-One-Out
MSCMs: Modified Shrinking Core Models
RCFs: Recycled Concrete Fines
RCP: Recycled Cement Paste
SCMs: Supplementary Cementitious Materials
SEM: Scanning Electron Microscope
SSA: Specific Surface Area
TGA: Thermogravimetric Analysis
XRD: X-ray Diffraction
XRF: X-ray Fluorescence
**Latin Symbols**
A: Pre-exponential factor, [m^2^·s^–1^]
CaO: CaO mass fraction in the RCFs, [wt.%]
*C*_0_: Dissolved CO_2_ concentration in water, [mol·m^–3^]
D: Effective diffusion coefficient through the product layer, [m^2^·s^–1^]
*D*_0_: Intrinsic diffusion coefficient through the product layer, [m^2^·s^–1^]
*d*_p0_: Initial diameter of particles, [m]
*E*_*a*_: Activation energy, [J·mol^–1^]
*f*_CaO_: Mass fraction of CaO in RCFs available for carbonation, [wt.%]
*k*_a_: Apparent reaction coefficient, [s^–1^]
*M*_CaO_: Molar mass of CaO, [kg mol^–1^]
MgO: MgO mass fraction in the RCFs, [wt.%]
N: Number of sampling points after carbonation reaction starts, [-]
n: Time index for the growth of the product layer, [-]
*P*_CO_2__: Partial pressure of CO_2_, [bar]
*SO*_3_: SO_3_ mass fraction in the RCFs, [wt.%]
T: Reaction temperature, [K]
*T*_ref_: Reference reaction temperature, [K]
*Th*_CO_2__: Theoretical potential of carbonation, [wt.%]
t: Reaction time in modeling, [s]
*t*_*s*_*p*__: Sampling time at point p during carbonation, [s]
*X*(*t*): Carbonation efficiency at reaction time t, [-]
**Greek Symbols**
α­(*t*): CO_2_ uptake at reaction time *t*, [-]
Δ*m*_*t*,30–975_: Mass loss between 30 and 975 °C, for normalizing data, [wt.%]
Δ*m*_*t*,500–900_: Mass loss between 500 and 900 °C, corresponding to CO_2_ content, [wt.%]
θ: Decay ratio, [-]
ρ: Density of the RCFs, [kg·m^–3^]
ρ_CaCO_3__: Molar density of the reaction product, [mol·m^–3^]
