# Supplementary material for: Wet Carbonation of Industrial Recycled Concrete Fines: Experimental Study and Reaction Kinetic Modeling
Source: Ind Eng Chem Res. 2025 Oct 30;64(45):21412–25. doi: 10.1021/acs.iecr.5c02835 (PMC12616853; doi:10.1021/acs.iecr.5c02835)
Supplement: Supplementary file 1 [file ie5c02835_si_001.pdf]

Supporting Information

Wet Carbonation of Industrial Recycled Concrete Fines: Experimental Study and Reaction Kinetic Modeling

Z. Tabrizi [1,2], C. Rodriguez [3,2], E. Barbera [1], W. R. Leal da Silva [2], F. Bezzo [1*]

[1] CAPE-Lab – Computer-Aided Process Engineering Laboratory, Department of Industrial Engineering, University of Padova, 35131 Padova, Italy.

[2] FLSmidth Cement A/S, Green Innovation, 2500 Valby, Copenhagen, Denmark.

[3] Institute for Technical Chemistry, Karlsruhe Institute of Technology, 76344 Karlsruhe, Germany.

* fabrizio.bezzo@unipd.it

S1. Formulation of models

S1.1. 1-Dimentional growth (plate shaped particles)

To describe the progression of wet carbonation in plate-shaped particles of an alkaline-earth metal (e.g., Ca or Mg), we consider an initial particle of thickness d_{p0}. As the reaction proceeds, a carbonated product layer forms and grows inward from the particle surfaces. Let x be the thickness of this product layer at time t. Consequently, the thickness of the remaining unreacted core is $d_{p0} - 2x$, as shown schematically in Fig. 1S(a). Hence,

$$d_p = d_{p0} - 2x \tag{S1}$$

Defining the reaction progress (or carbonation efficiency) X as the volumetric fraction of reacted material (i.e., the ratio of reacted volume to the initial particle volume), we obtain:

$$1 - X = \frac{A_s \cdot (d_{p0} - 2x)}{A_s \cdot d_{p0}} = \frac{d_{p0} - 2x}{d_{p0}} = 1 - \frac{2x}{d_{p0}} \tag{S2}$$

which simplifies to

$$x = \frac{d_{p0} \cdot X}{2} \tag{S3}$$

Differentiating with respect to X yields:

$$\frac{dx}{dX} = \frac{d_{p0}}{2} \tag{S4}$$

For solid-state diffusion over extended time periods, a parabolic rate law is often more accurate than a linear (Fickian) treatment [1]. Parabolic rate laws have been successfully employed for metal oxidation processes, which share mechanistic similarities with mineral carbonation (i.e., formation of thick product layers). In this framework, the depth of the reacted layer x follows:

$$x = K \cdot t^n \tag{S5}$$

where K is a growth-rate coefficient, and n is a time exponent. For volume diffusion–controlled reactions, n has been reported to be approximately 0.5, which leads directly to the classical parabolic growth law. Substituting $n = 0.5$ into Eq. (S5) yields:

$$x^2 = K^2 \cdot t = \frac{2 \cdot C_0 \cdot D \cdot t}{\rho_{CaCO_3}} \tag{S6}$$

where C_0 is the concentration of dissolved CO_2 (from Eq. (6)), ρ_{CaCO_3} is the molar density of the reaction product, and D is the effective diffusion coefficient through the product layer. Differentiating Eq. (S6) with respect to time gives:

$$\frac{dx}{dt} = \frac{2 \cdot C_0 \cdot D}{\rho_{CaCO_3} \cdot x} \tag{S7}$$

As the product layer thickens, the diffusivity typically diminishes due to pore clogging and changes in porosity. Therefore, D is expressed as a dynamic term $D_0 \cdot \varphi$, where D_0 is the intrinsic diffusion coefficient through the product layer of naturally carbonated material, and φ is a ratio which decreases from 1 to 0 during the reaction:

$$D = D_0 \cdot \varphi \tag{S8}$$

Substituting into Eq. (S7) yields:

$$\frac{dx}{dt} = \frac{2 \cdot C_0 \cdot D_0 \cdot \varphi}{\rho_{CaCO_3} \cdot x} \tag{S9}$$

Because D_0 itself can be expressed as an Arrhenius-type term (Eq. (7)), we combine these relations with the definition of x in terms of X (Eqs. (S3) and (S6)) to express the rate of change of X:

$$\frac{dX}{dt} = \frac{4 \cdot C_0 \cdot D_0 \cdot \varphi}{\rho_{CaCO_3} \cdot d_{p0}^2 \cdot X} = \frac{k_a \cdot \varphi}{X} \tag{S10}$$

where:

$$k_a = \frac{4 \cdot C_0 \cdot D_0}{\rho_{CaCO_3} \cdot d_{p0}^2} \tag{S11}$$

The time dependence of φ accounts for the decrease in pore connectivity, decay ratio (θ) and effective diffusivity as the carbonated layer thickens [2]. Its rate of change is described by:

$$-\frac{d\varphi}{dt} = k_a \cdot \theta \cdot \varphi \tag{S12}$$

which can be integrated to give:

$$\varphi = exp(-k_a \cdot \theta \cdot t) \tag{S13}$$

Substituting Eq. (S13) into Eq. (S10) leads to the final ordinary differential equation that governs the one-dimensional growth of the carbonated product layer:

$$\frac{dX}{dt} = \frac{k_a \cdot exp(-k_a \cdot \theta \cdot t)}{X} \tag{S14}$$

subject to the initial condition $X(t = 0) = 0$.

S1.2. 3-Dimensional Growth (Spherical shaped Particles)

For spherical shaped particles we consider an initial particle radius of ${d_{p0}}/{2}$, where x represents the thickness of the carbonated product layer at time t. Consequently, the radius of the remaining unreacted core is ${d_{p0}}/{2} - x$, as illustrated schematically in Fig. 1S(b). The relationship between the particle radii is given by:

$$\mathrm{d_p}/2 = \mathrm{d_{p0}}/2 - x \tag{S15}$$

Defining the reaction progress X as the volumetric fraction of reacted material (i.e., the ratio of reacted volume to the initial particle volume), we obtain:

$$1 - X = \frac{4/3 \cdot \pi \cdot (\mathrm{d_{p0}}/2 - x)^3}{4/3 \cdot \pi \cdot (\mathrm{d_{p0}}/2)^3} = \frac{(\mathrm{d_{p0}}/2 - x)^3}{(\mathrm{d_{p0}}/2)^3} = (1 - \frac{2 \cdot x}{\mathrm{d_{p0}}})^3 \tag{S16}$$

Simplifying, this becomes:

$$x = \frac{d_{p0}}{2}\left[1 - (1 - X)^{1/3}\right] \tag{S17}$$

Differentiating x with respect to X we have:

$$\frac{dx}{dX} = \frac{d_{p0}}{6} \cdot (1 - X)^{-2/3} \tag{S18}$$

The remainder of the derivation follows the same principles as for plate-shaped particles. Substituting the expression for x and $\frac{dx}{dX}$, the final model for spherical particles is:

$$\frac{dX}{dt} = \frac{k_a \cdot exp(-k_a \cdot \theta \cdot t)}{(1 - (1 - X)^{1/3}) \cdot (1 - X)^{-2/3}} \tag{S19}$$

where the apparent reaction coefficient k_a is:

$$k_a = \frac{12 \cdot C_0 \cdot D_0}{\rho_{CaCO_3} \cdot d_{p0}^2} \tag{S20}$$

Equations (S14, and S19) show that carbonation kinetics are driven by the rate coefficient k_1 but become increasingly limited over time by the exponentially decaying factor θ. This formulation captures both the initial rapid reaction (when the product layer is thin and $(\varphi \approx 1)$ and the subsequent slowing as diffusion pathways through the product layer become more tortuous (when φ decreases).

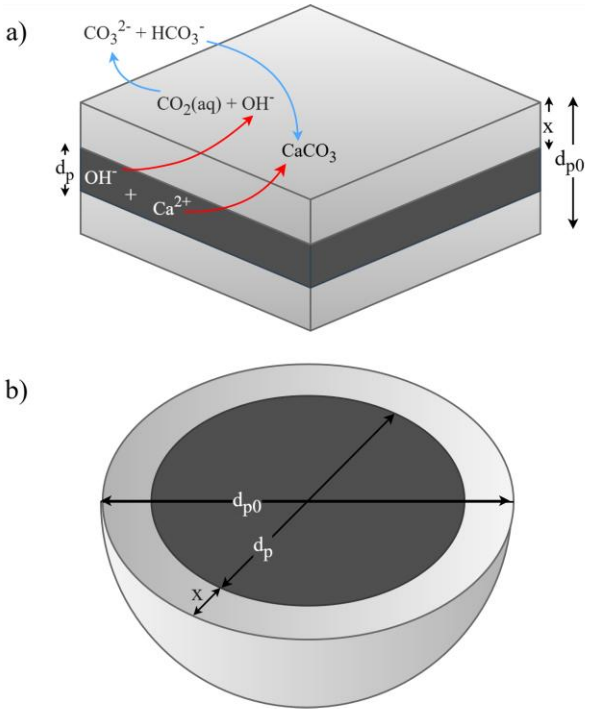

Figure S1. Schematic representation of (a) plate, and (b) spherical particle shapes during carbonation.

S2. Model Identification approach

S2.1. Lest Square method

The least squares method is a statistical approach used to estimate parameters in a model by minimizing the sum of the squared differences between observed and predicted values [3]. Given a dataset with observed values y_i and predicted values $\hat{y}_i$, the objective is to minimize the residual sum of squares (RSS), defined as:

$$RSS = \sum_{i=1}^{n} (y_i - \hat{y}_i)^2 \quad \text{(S21)}$$

This method ensures the best possible fit by reducing the overall error in the model. The goodness of fit is often quantified using the coefficient of determination (R^2), which measures how well the model explains the variance in the observed data. It is calculated as:

$$R^2 = \sum_{i=1}^{n} \frac{RSS}{TSS} \tag{S22}$$

where TSS (total sum of squares) is:

$$TSS = \sum_{i=1}^{n} (y_i - \bar{y})^2 \tag{S23}$$

and $\bar{y}$ is the mean of the observed values. An R^2 value close to 1 indicates a strong correlation between the model and the observed data, suggesting high predictive accuracy.

S2.2. Model discrimination with P-test

Model discrimination using the P-test is a statistical approach to determine the most reliable model among competing candidates by evaluating their goodness-of-fit. This method assigns a relative probability (P_i) to each model, representing the likelihood of it being the best descriptor of the system [4]. The calculation is based on the chi-square (χ^2) values obtained after parameter estimation, where lower chi-square values indicate better model performance. The probability of the i-th model, is given by:

$$P_i = \frac{1/\chi_i^2}{\sum_{i=1}^{N_M} 1/\chi_i^2} \cdot 100 \tag{S24}$$

where N_M is the total number of candidate models. This approach ranks models based on their ability to minimize discrepancies between observed and predicted data. A higher P_i value for one model provides strong evidence of its superiority, ensuring effective discrimination.

S2.3. Model precision with t-test

The t-test for parameter precision was employed to evaluate the reliability of estimated parameters by assessing their statistical significance relative to their associated uncertainties. Since parameter estimation involves uncertainty due to measurement noise and model assumptions, each estimated parameter $\hat{\theta}_i$ follows a probability distribution. The t-statistic was computed to quantify the precision of each parameter, ensuring that it was well-determined within the confidence interval [5].

The t-value for each parameter was calculated as:

$$t_i = \frac{\hat{\theta}_i}{\sqrt{v_{\theta,ii}}}, \qquad i = 1, \cdots, N_\theta \tag{S25}$$

where $\hat{\theta}_i$ is the estimated parameter, and CI_i is the 95% confidence interval, determined as:

$$\mathrm{CI}_i = \sqrt{v_{\theta,ii}} \times t(\frac{1-0.05}{2}, DoF), \qquad i = 1, \cdots, N_\theta \tag{S26}$$

Here $v_{\theta,ii}$ represents the diagonal element of the covariance matrix, corresponding to the variance of parameter $\hat{\theta}_i$, while $t(95\%, DoF)$ denotes the reference t-value for a 95% confidence level, derived based on the number of degrees of freedom (DoF).

A higher t-value indicated that the parameter was estimated with greater precision, implying a narrower probability distribution and lower uncertainty. The computed t-values were compared against critical reference values to determine whether each parameter was sufficiently well-defined within the confidence bounds.

S3. Model Identification of RCP carbonation

Following the same workflow, presented at Fig. 2 and employed to identify a model for RCF carbonation data, a preliminary estimation of parameters was conducted to provide the estimates and uncertainty in estimation (Fig. 2, Step 1). This data is presented at Table S1 for the set of candidate models (Table 1), and shows the high uncertainty in estimation of A, the 95% t-values to be compared with reference t-value of this system, which is 2.16. Preliminary estimation of parameters also indicate the higher predictability of the $MSCM_{f,c}$, and $MSCM_{p,c}$, models, which are based on a 2D growth mechanism for cylindrical mineral particles.

Table S1. Parameter estimates and t-values for RCP carbonation candidate models.

	$\boldsymbol{A}$		$\boldsymbol{E_a}$		$\boldsymbol{\theta}$		
**Models**	**Estimation [m^2.s^{-1}]**	**t-value**	**Estimation [kJ.mol^{-1}]**	**t-value**	**estimation**	**t-value**	$\boldsymbol{R^2}$
$BSCM_{f,c}$	6.1209E-9	0.7104	22327	5.9890	-	-	0.9187
$BSCM_{f,s}$	3.4241E-9	0.7570	22591	6.4576	-	-	0.9378
$MSCM_{f,p}$	2.7083E-8	1.5095	24145	13.7893	1.9312	25.2265	0.9942
$MSCM_{f,c}$	7.7843E-9	1.7777	24174	16.2894	5.2564	20.5784	0.9959
$MSCM_{f,s}$	3.3054E-9	1.6731	24170	15.3118	13.6216	22.3049	0.9954
$MSCM_{p,p}$	4.8012E-8	1.5095	24144	13.7887	3.8624	25.2260	0.9942
$MSCM_{p,c}$	1.4890E-8	1.9684	24191	18.0714	4.1206	17.6377	0.9967
$MSCM_{p,s}$	2.9390E-9	0.8571	22964	7.4390	9.9853E-7	1.559E-4	0.9648

To evaluate the estimability of the parameters (Fig. 2, Step 2), an EA-Orthogonalisation method was employed. Table S2 presents the results for parameter ranking and subset selection. In most models, the decay ratio (θ) is identified as the most relevant parameter, while pre-exponential factor (A) and activation energy (E_a) show comparable relevance, becoming partially important in some cases. This observation aligns with the reported formation of a denser product layer around carbonated RCP, which increases the significance of the decay ratio.

Table S2. Estimability analysis results for different candidate models.

	A		E_a		θ	
**Models**	**Rank**	**Selected**	**Rank**	**Selected**	**Rank**	**Selected**
$BSCM_{f,c}$	2	yes	1	yes	—	—
$BSCM_{f,s}$	2	yes	1	yes	—	—
$MSCM_{f,p}$	3	no	2	no	1	yes
$MSCM_{f,c}$	3	no	2	no	1	yes
$MSCM_{f,s}$	2	yes	3	no	1	yes
$MSCM_{p,p}$	3	no	2	no	1	yes
$MSCM_{p,c}$	2	no	3	no	1	yes
$MSCM_{p,s}$	2	yes	1	yes	3	no

Table S3 presents the estimation and uncertainty of kept parameters, together with P-values for discrimination between models (Fig. 2, Step 3). Based on the obtained results, even after refinements, Models $BSCM_{f,c}$, $BSCM_{f,s}$, and $MSCM_{p,s}$ were not precisely identified. Model $MSCM_{p,c}$, with a higher P-value, was selected as the most representative model for the system. The effect of model simplification in increasing prediction precision compared to experimental data is depicted in Fig. S2. This was further validated using the same methodology (Fig. 2, Step 4), yielding a high validation R^2 of 0.9966 (±0.0017).

Table S3. Parameter estimates and P-values for RCP carbonation candidate models after screening.

	A		E_a		θ		
**Models**	**Estimation [$m^2.s^{-1}$]**	**t-value**	**Estimation [$J.mol^{-1}$]**	**t-value**	**estimation**	**t-value**	**p-value**
$BSCM_{f,c}$	6.1272E-9	0.7104	22329	5.9832	-	-	0.0675
$BSCM_{f,s}$	3.4244E-9	0.7566	22591	6.4546	-	-	0.1244
$MSCM_{f,p}$	-	-	-	-	1.9312	32.6873	13.6333
$MSCM_{f,c}$	-	-	-	-	5.2564	27.7138	22.7818
$MSCM_{f,s}$	3.3054E-9	21.1710	-	-	13.6221	23.4182	19.0496
$MSCM_{p,p}$	-	-	-	-	3.8624	32.6873	13.6325
$MSCM_{p,c}$	-	-	-	-	4.1205	24.4961	30.2820
$MSCM_{p,s}$	2.9408E-9	0.9008	22966	7.8052	-	-	0.4288

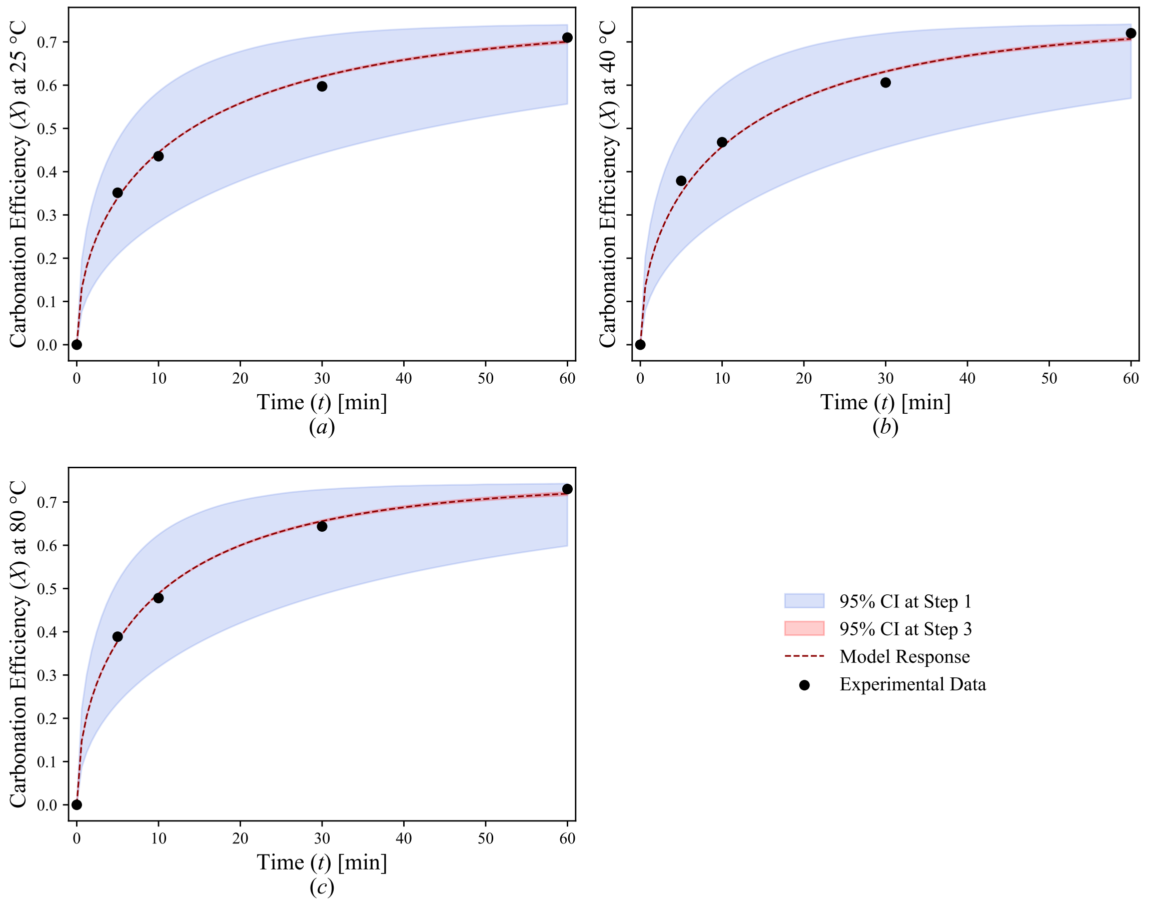

Figure S2. Experimental data and Representative model response with 95% CI for the original model at Step 1 and the model at Step 3 after fixing pre-exponential factor and activation energy for: (a) 25, (b) 40, and (c) 80 °C. The coloured area represents the impact of parametric uncertainty on model predictions.

S3. Supplementary characterisation results

The complete set of X-ray diffraction (XRD) patterns for RCFs and cRCFs at 180 min (i.e., the end of the reaction) from different experimental batches and temperatures are shown in Fig. S3. The Particle Size Distributions (PSDs) of RCFs during the reaction at 25 °C and 65 °C are presented in Fig. S4, which served as the basis for selecting the samples for accumulated PSDs shown in Fig. 3. Thermogravimetric analysis (TGA) results for RCFs, cRCFs at 10 min of reaction, and the fine fraction of cRCFs (screened to below 22 μm) at 10 min are shown in Fig. S5. Attenuated Total Reflection (ATR) spectra of RCFs, and cRCFs at the different temperatures are shown in Fig. S6.

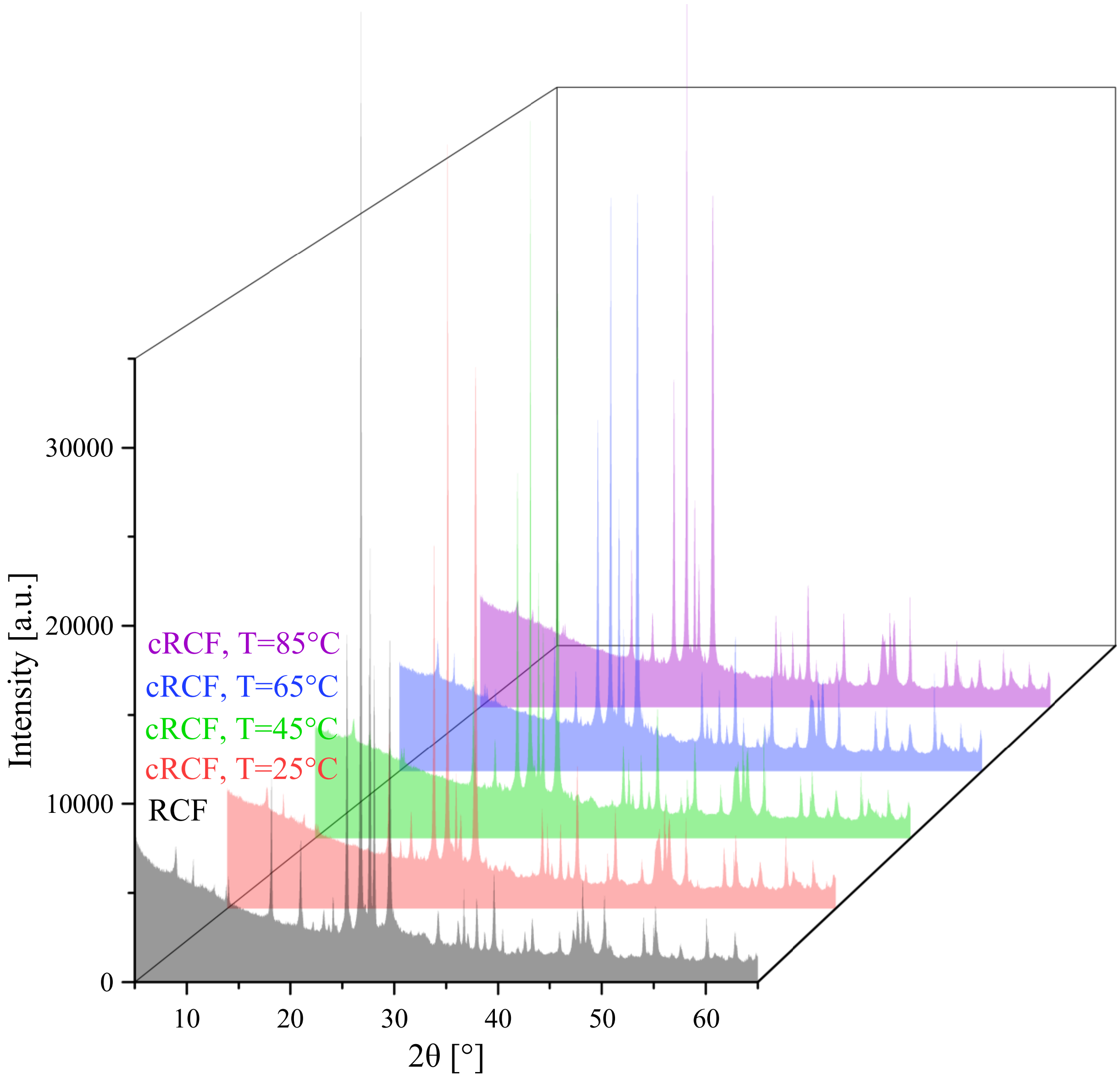

Figure S3. X-ray diffraction patterns of the RCFs and cRCFs at 25, 45, 65, and 85 °C.

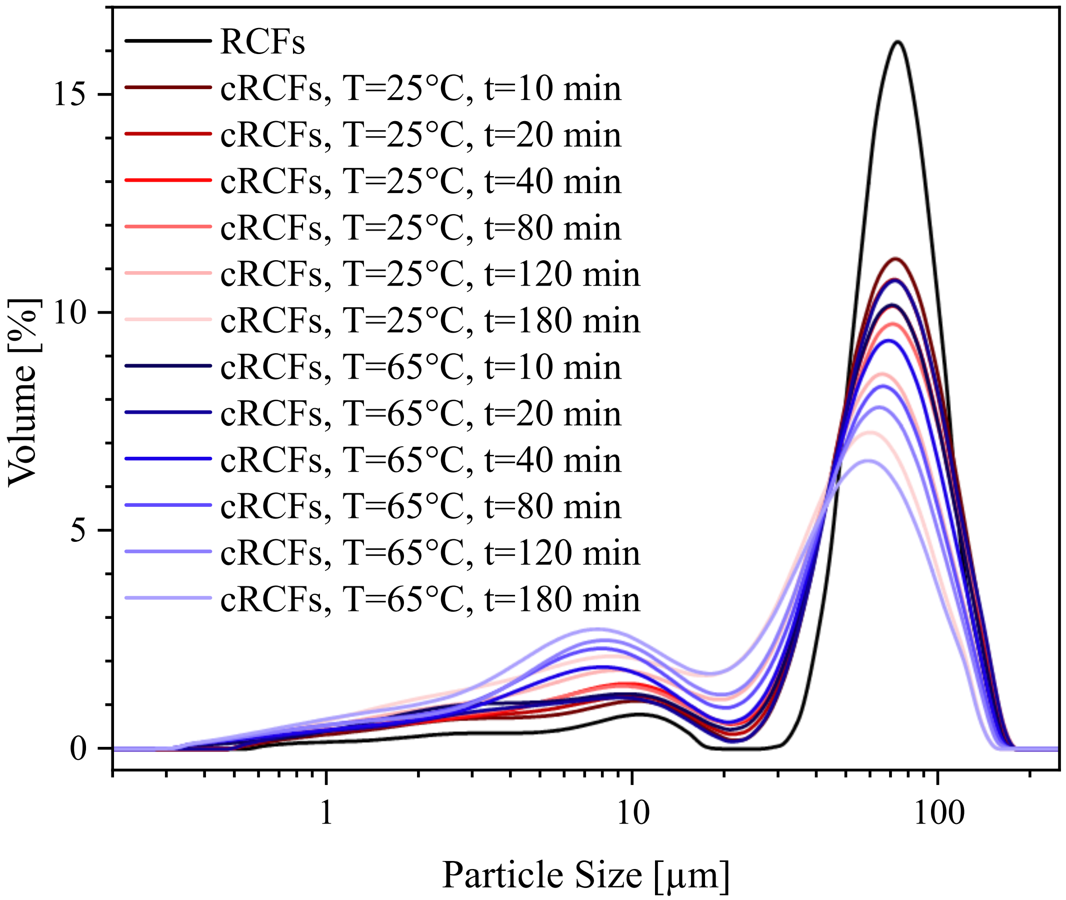

Figure S4. Particle size distribution for the RCFs, and cRCFs at 65°C and 25°C during reaction.

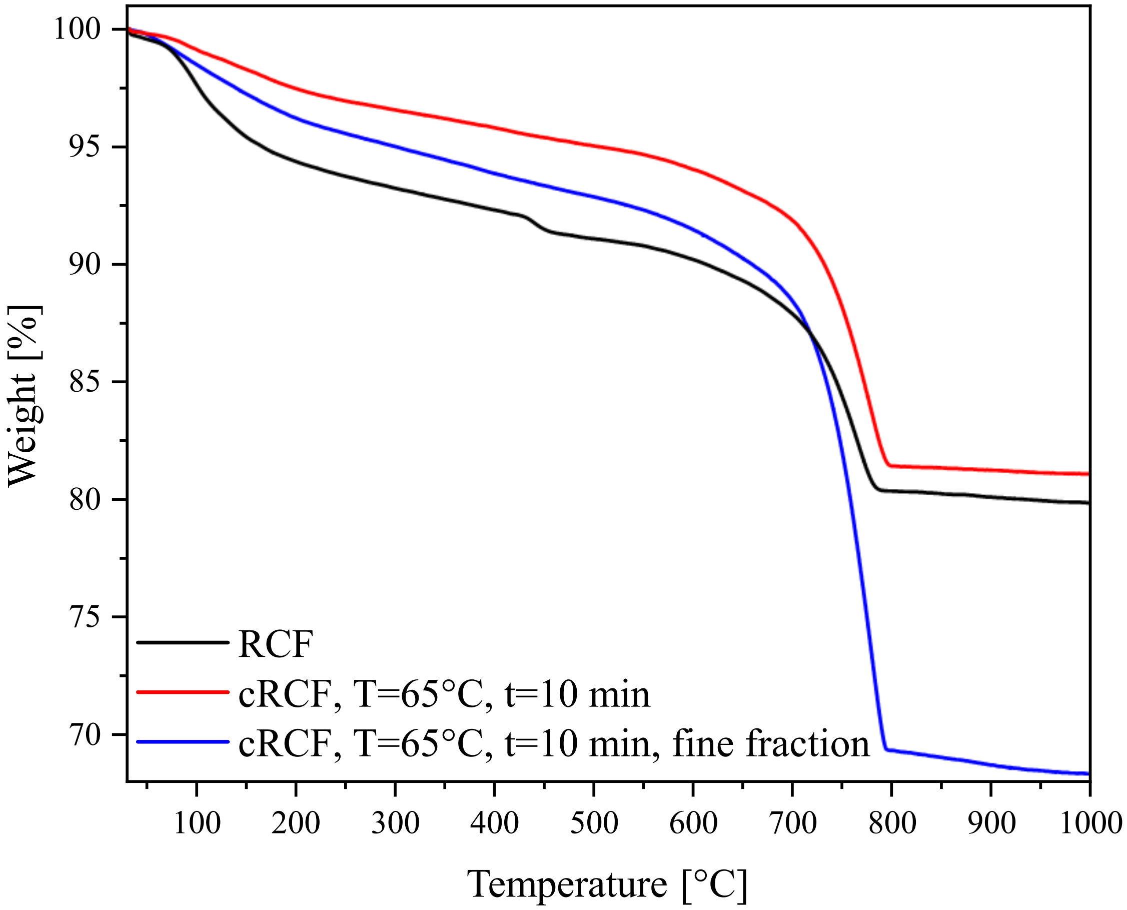

Figure S5. TGA results of the starting RCF and cRCF after 10 minutes of reaction at 65 °C, shown for both the whole product and the fine fraction (<20 μm).

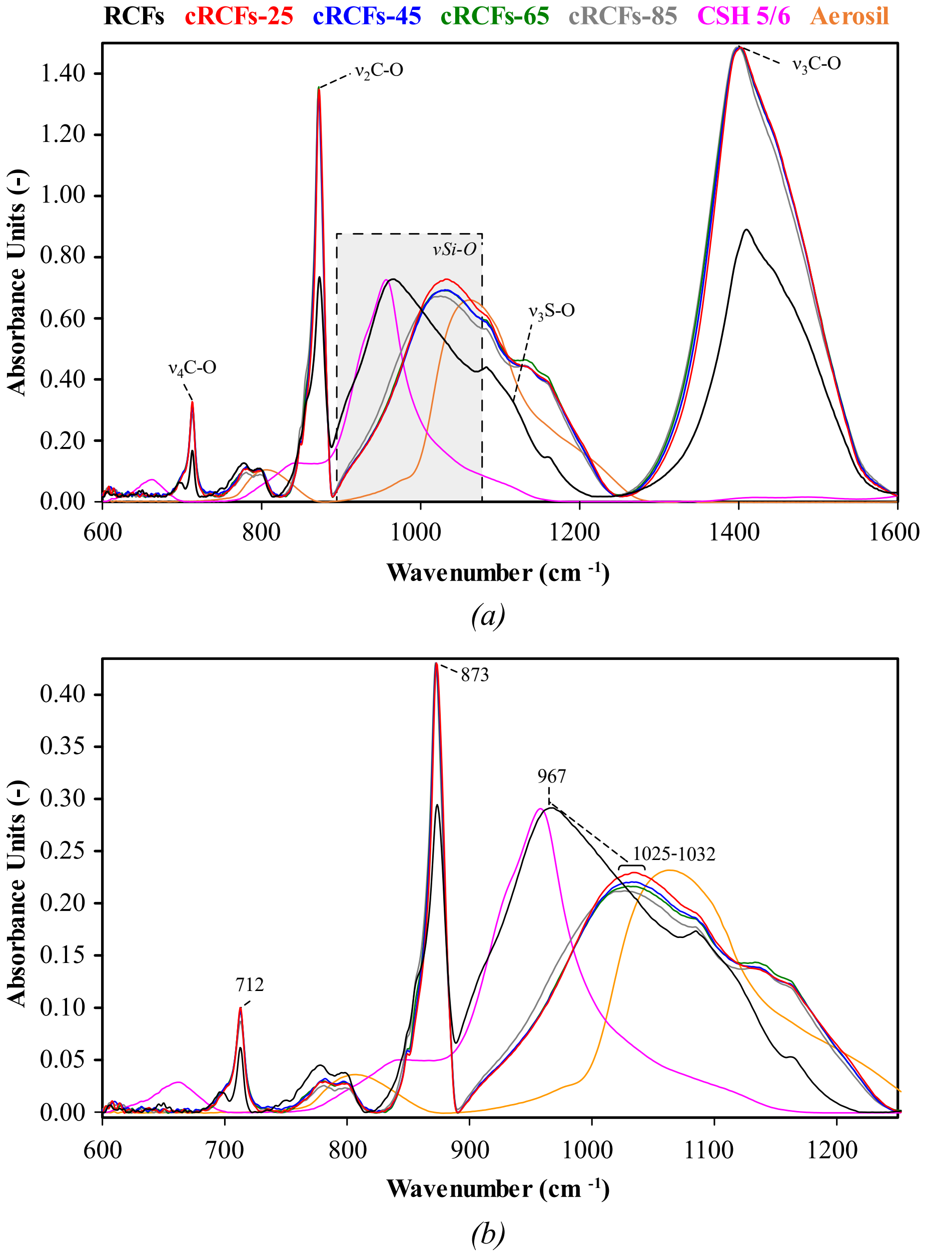

Figure S6. ATR spectra of RCFs, and cRCFs at the different temperatures. Pure components are also shown as references: C-S-H (C/S =5/6) and amorphous highly dispersed SiO_2 (Aerosil). The ν_3(Si–O) band shifts from 967 cm^{-1} in the RCFs to 1025–1032 cm^{-1}in the cRCFs indicating increased silicate polymerisation during carbonation since it is reported the shift to higher wave number[6,7] , Similarly, the ATR spectra confirm carbonates presence by the absorption bands at 713, 873, and 1390–1405 cm^{-1}.

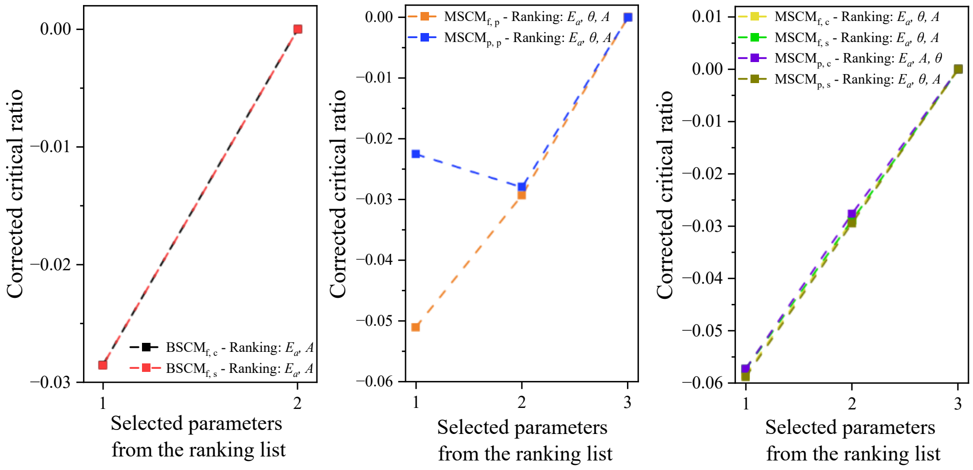

(a) *(b)* *(c)*

Figure S7. Effect of number of parameters estimated on corrected critical ratio of models: (a) basic Shrinking Core Models with 2 parameters, (b) most predictive model after preliminary estimation, and (c) rest of the modified Shrinking Core Models.

Table S4. Variations of CO_2 solubility and diffusion coefficient with temperature at the end of the carbonation reaction of RCFs.

Temperature (°C)	Diffusion Coefficient (D) [$m^2.s^{-1}$]	CO_2 Solubility (C_0) [$mmol.L^{-1}$]
25	7.95E-12	5.25
45	1.29E-11	3.165149
65	1.98E-11	2.025934
85	2.89E-11	1.363005

Nomenclature

Latin Symbols

A	Pre-exponential factor, [$m^2 \cdot s^{-1}$]
A_s	Surface area of the plate-shaped particle, [m^2]
C_0	Dissolved CO_2 concentration in water, [$mol \cdot m^{-3}$]
D	Effective diffusion coefficient through the product layer, [$m^2 \cdot s^{-1}$]
D_0	Intrinsic diffusion coefficient through the product layer, [$m^2 \cdot s^{-1}$]
d_{p0}	Initial diameter of particles, [m]
d_p	Diameter of the unreacted particle core, [m]
E_a	Activation energy, [$J \cdot mol^{-1}$]
k_a	Apparent reaction coefficient, [s^{-1}]
K	Growth-rate coefficient, [-]
n	Time index for the growth of the product layer, [-]
T	Reaction temperature, [K]
t	Reaction time, [s]
$X(t)$	Carbonation efficiency, [-]

Greek Symbols

θ	Decay ratio, [-]
ρ_{CaCO_3}	Molar density of the reaction product, [$mol \cdot m^{-3}$]
φ	Ratio of the effective diffusion coefficient to the intrinsic diffusion coefficient, [-]

References

(1) Xu, Z.; Rosso, K. M.; Bruemmer, S. M. A Generalized Mathematical Framework for Thermal Oxidation Kinetics. *J Chem Phys* **2011**, *135* (2).

(2) Miao, E.; Du, Y.; Wang, H.; Xiong, Z.; Zhao, Y.; Zhang, J. Experimental Study and Kinetics on CO2 Mineral Sequestration by the Direct Aqueous Carbonation of Pepper Stalk Ash. *Fuel* **2021**, *303*, 121230.

(3) Bard, Y. *Nonlinear Parameter Estimation*; Academic Press, 1974.

(4) Galvanin, F.; Cao, E.; Al-Rifai, N.; Gavriilidis, A.; Dua, V. A Joint Model-Based Experimental Design Approach for the Identification of Kinetic Models in Continuous Flow Laboratory Reactors. *Comput Chem Eng* **2016**, *95*, 202–215.

(5) Franceschini, G.; Macchietto, S. Model-Based Design of Experiments for Parameter Precision: State of the Art. *Chem Eng Sci* **2008**, *63* (19), 4846–4872.

(6) García Lodeiro, I.; Macphee, D. E.; Palomo, A.; Fernández-Jiménez, A. Effect of Alkalis on Fresh C-S-H Gels. FTIR Analysis. *Cem Concr Res* **2009**, *39* (3), 147–153.

(7) Ashraf, W.; Olek, J. Carbonation Behavior of Hydraulic and Non-Hydraulic Calcium Silicates: Potential of Utilizing Low-Lime Calcium Silicates in Cement-Based Materials. *J Mater Sci* **2016**, *51* (13), 6173–6191.
